# From energy to cellular forces in the Cellular Potts Model: An algorithmic approach

**DOI:** 10.1371/journal.pcbi.1007459

**Published:** 2019-12-11

**Authors:** Elisabeth G. Rens, Leah Edelstein-Keshet

**Affiliations:** Department of Mathematics, University of British Columbia, Vancouver, British Columbia, Canada; University at Buffalo - The State University of New York, UNITED STATES

## Abstract

Single and collective cell dynamics, cell shape changes, and cell migration can be conveniently represented by the Cellular Potts Model, a computational platform based on minimization of a Hamiltonian. Using the fact that a force field is easily derived from a scalar energy (**F** = −∇*H*), we develop a simple algorithm to associate effective forces with cell shapes in the CPM. We predict the traction forces exerted by single cells of various shapes and sizes on a 2D substrate. While CPM forces are specified directly from the Hamiltonian on the cell perimeter, we approximate the force field inside the cell domain using interpolation, and refine the results with smoothing. Predicted forces compare favorably with experimentally measured cellular traction forces. We show that a CPM model with internal signaling (such as Rho-GTPase-related contractility) can be associated with retraction-protrusion forces that accompany cell shape changes and migration. We adapt the computations to multicellular systems, showing, for example, the forces that a pair of swirling cells exert on one another, demonstrating that our algorithm works equally well for interacting cells. Finally, we show forces exerted by cells on one another in classic cell-sorting experiments.

## Author summary

Cells exert forces on their surroundings and on one another. In simulations of cell shape using the Cellular Potts Model (CPM), the dynamics of deforming cell shapes is traditionally represented by an energy-minimization method. We use this CPM energy, the Hamiltonian, to derive and visualize the corresponding forces exerted by the cells. We use the fact that force is the negative gradient of energy to assign forces to the CPM cell edges, and then extend the results to approximate interior forces by interpolation. We show that this method works for single as well as multiple interacting model cells, both static and motile. Finally, we show favorable comparison between predicted forces and real forces measured experimentally.

## Introduction

From embryogenesis and throughout life, cells exert forces on one another and on their surroundings. Cell and tissue forces drive cell shape changes and cell migration by regulating cell signaling and inducing remodeling of the cytoskeleton. Along with progress in experimental quantification of cellular forces, there has been much activity in modeling and developing computational platforms to explore cellular mechanobiology. In some platforms, among them vertex-based and cell-center based simulations, the shape of a cell is depicted by convex polygons, ellipsoids or spheres.

The Cellular Potts Model (CPM) is a convenient and relatively popular computational platform for modeling dynamic, irregular and highly fluctuating cell shapes [1–3]. An advantage of the CPM is its high resolution description of cell shapes compared with polygonal cells in vertex-based computations [4]. The CPM can easily accommodate cell detachment or reattachment from an aggregate, and a range of cell-cell adhesion, where vertex-based simulations are less suitable. The CPM also captures stochastic aspects of cell movement and deformation. At the same time, since CPM computations are based on a phenomenological “energy” (the Hamiltonian), it has often been criticized as non-physical, or, at least, as devoid of Newtonian forces. For a detailed rebuttal of this issue, see the recent work of [5].

In their review of models for cell migration, Sun and Zaman [6] point to the need to coordinate results between force-based and energy-based models, indicating that this is a “challenging but significant” problem. Here we devise a map between the CPM Hamiltonian and an explicit vector-field of forces associated with the dynamics of cell shape. Our approach contrasts with that of [7, 8] who used the CPM to describe cell shape, but who assumed phenomenological force-fields unrelated to the underlying Hamiltonian. In [9, 10], analytical expressions for forces obtained from a specific Hamiltonian function were employed. While CPM forces were discussed in detail in [5], those forces were quantified explicitly for simple geometries, such as circular or spherical cells. The algorithm we describe here computes force-fields that are consistent with an arbitrary Hamiltonian and cell shapes, links those forces to a typical internal signaling computation, and generalizes to multiple interacting cells. We illustrate the computation of the force field for single cells exerting traction forces on a 2D substrate, for pairs of cells pulling/pushing one another, and for larger clusters of cells interacting through adhesion and through internal signaling.

In the Cellular Potts model, each “cell” configuration *σ*, consists of a collection of connected lattice site, assigned a unique index (“spin number”). Parts of the domain containing no cells are indexed 0 by convention. For a single CPM cell surrounded by medium, the typical Hamiltonian is given by

$$\begin{array}{c} H\left(\sigma \right)=H_{A}+H_{P}+H_{J}, \end{array}$$ (0.1)

where *σ* is the cell configuration and

$$\begin{array}{c} H_{A}=\lambda_{a}{\left(A-a\right)}^{2},\;H_{P}=\lambda_{p}{\left(P-p\right)}^{2},\;H_{J}=J\left(0,1\right)P. \end{array}$$ (0.2)

Here *H*_*A*_ is an energetic cost for expansion or contraction of the area, *A*, away from a constant “rest area”, *a*, of the cell. *H*_*P*_ is an energetic cost for deviation of the cell perimeter *P* from its “rest perimeter” *p*. *H*_*J*_ is an energy associated with the cell-medium interface (generalized later to include cell-cell or cell-medium adhesive energies.) The factors λ_*a*_, λ_*p*_, *J*(0, 1) set the relative energetic costs of area changes, perimeter changes, and changes in the contact with the medium. In a typical CPM simulation, cell shapes are highly deformable. At each simulation step (Monte Carlo Step, MCS) every boundary pixel of each cell may “protrude” or “retract”. Due to the historical connection to the Ising model [11], these changes are sometimes called “spin-flips”. The pixel changes are accepted or rejected with some probability that depends on the change in *H* and on a user-defined “temperature” *T*, as described in Materials and Methods.

There are many realizations of the Potts Model with additional terms, or variations of such terms. In the Discussion, we summarize the numerous ways that CPM cell shape computations were linked to force calculations external to the CPM formalism itself (and including, among others, finite element methods).

Since the Hamiltonian associates an “energy” with each cellular configuration, theoretically we can relate forces to the negative gradient of the Hamiltonian, i.e.

$$\begin{array}{c} \vec{F}=(F_{x},F_{y})=-\nabla H=-(\frac{\partial H}{\partial x},\frac{\partial H}{\partial y}). \end{array}$$ (0.3)

**Fig 1:**
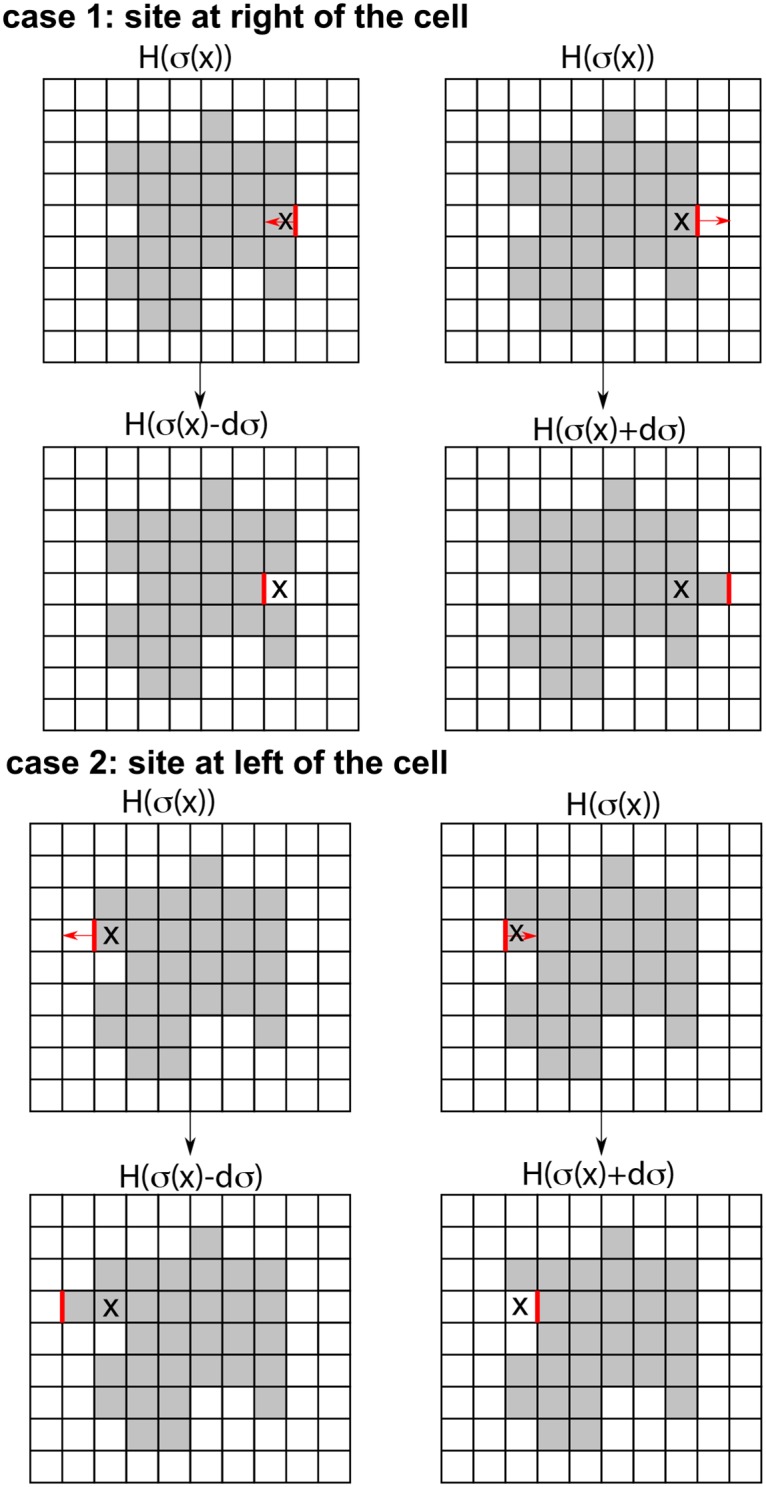
Schematic diagram: Deriving forces from a Cellular Potts Model Hamiltonian. The Hamiltonian represents an energy, so $\vec{F}=-\nabla H$. We compute a discrete approximation to the components of the force (*F*_*x*_, *F*_*y*_) at each point $\vec{x}$ on the cell boundary. Centered finite differences are used to approximate the derivatives −(∂*H*/∂*x*, ∂*H*/∂*y*) of the Hamiltonian as in Eq (0.5). Here we illustrate the idea for the *x* component of the force, *F*_*x*_. From a given initial CPM cell configuration *σ* (top row), we numerically compute the difference in the Hamiltonian at a point $\vec{x}$ on the right cell boundary when the cell retracts or extends (second row). We show the same idea for the left cell boundary (last two rows). The force field computed along the boundary is then smoothed and interpolated to the cell interior, as described in Materials and Methods.

In practice, the computations are all carried out on a finite grid, so partial derivatives in (0.3) are approximated by finite differences. Viewing cells on a 2D substrate from above, for example, we calculate the small change in the Hamiltonian when the cell boundary is extended in the *x* or *y* directions by a small step Δ*x* or Δ*y*, as illustrated in Fig 1. This is repeated at each point along the edge of the cell.

Although the CPM can only directly prescribes forces on the edge of the cell, we use interpolation to approximate the force field in the the interior of the cell region (i.e., to visualize putative traction forces created by the cellular “footprint” on its substrate or extracellular matrix). In the absence of additional model refinements (e.g. model of evolving sites of focal adhesions, internal structures and/or actin stress fibers), we do so by simple linear interpolation from the cell edge to the cell centroid. This is a rough approximation that can be adapted or modified in future studies. The interpolation is validated against experimental data. (We compared linear, quadratic, and exponential fits to experimental data, showing that they lead to similar results, S5 Fig). The workflow then entails 1) calculating the force along the cell perimeter, 2) reducing the grid effect in the force field, 3) interpolating the force-field to the interior of the cell.

Visualizing the interior force field can serve several purposes. First, it is useful in modeling experimental cell traction forces, as we show further on. Second, it can be used in computational studies where such forces are linked to feedback between cell mechanics and intracellular signaling. Our method could provide a useful companion to computations in [7, 8], where the CPM was linked to a finite element model of cell-substrate forces, or to [12], where feedback between traction forces, cell shape and adhesions were modeled. This generic computation can be extended to forces of multiple interacting cells (in a cell sheet or aggregate). The implementation of this idea is described in the Materials and Methods, with further details in the Supporting Information S1 File.

## Results

### Forces associated with static cell shapes

**Fig 2:**
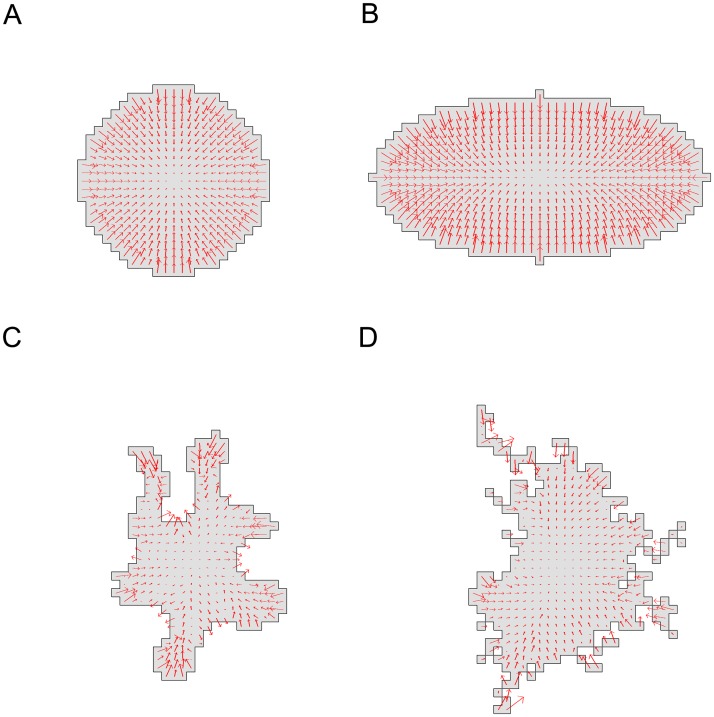
Forces predicted for several cell shapes. Force fields predicted by our complete method (smoothing and interpolation) for four simulated cell shapes in the CPM. (A) Circular cell (area *A* = 401, perimeter *P* = 74, diameter = 23). (B) Elliptical cell (area *A* = 629, perimeter *P* = 101, axes lengths 21 and 41). (C) Irregular cell shape (area *A* = 301, perimeter *P* = 118). (D) Highly irregular cell shape (area *A* = 400, perimeter *P* = 146). Parameter values were *a* = 300, λ_*a*_ = 10, *p* = 100, λ_*p*_ = 10, *J*(0, 1) = 3000, *ξ*(*r*) = 18, and *r* = 3 for all neighborhood calculations. We used a grid of 50 by 50 lattice sites with Δ*x* = 1. See also Supplementary Figures S6–S8 Figs for intermediate steps in the calculation of forces.

We computed the force-fields associated with the CPM Hamiltonians of single static cells with circular (A), elliptical (B), and irregular shapes (C,D). Results of the complete algorithm (including smoothing and interior forces) are shown in Fig 2(A)–2(D). Intermediate calculations (forces on the cell boundary without and with smoothing, and without interior smoothing) can be found in S6–S8 Figs.

Whether forces point inwards or outwards depends on the values of the area *A* and perimeter *P* relative to their target values *a*, *p* and the relative weights of the energetic cost or area and perimeter changes. For parameters given in Fig 2, forces point inwards all along the boundary of the circular and elliptical cell shapes. We find forces directed approximately normal to the boundary, with magnitudes that decay towards the centroid, as a consequence of our interpolation.

In more irregular shapes (Fig 2C and 2D), forces can point either inwards or outwards at different points along the boundary. For the irregular cell with given configuration and Hamiltonian parameters, we found that at convex sites, the forces point inwards, while at concave sites, the forces point outwards. This is also in line with expectations based on local (positive or negative) curvature of the boundary. Even for the most irregular cell shape, the forces are fairly smooth and continuous.

### Dynamic cell shapes and evolving forces

We next tracked the evolution of forces that accompany dynamic changes in shape of a CPM “cell”. To do so, we initiated a CPM computation with a circular cell with perimeter smaller than the rest-length *p* and area greater than the rest area, *a*. We also assumed λ_*p*_ > λ_*a*_, so that the energetic cost of the perimeter term dominated the energetic cost of the area term in the Hamiltonian.

**Fig 3:**
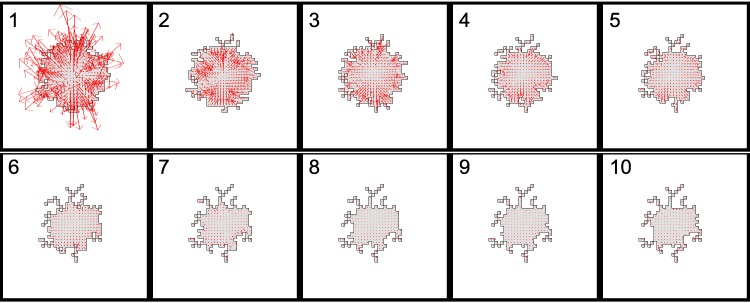
Dynamics of cell shape and the evolution of forces. Time series from 1 to 10 MCS. The cell achieves force balance by decreasing its area towards the rest area *a* and increasing its perimeter towards the rest perimeter *p*. Parameters were *a* = 200, λ = 8, *p* = 100, λ_*p*_ = 2000, *J*(0, 1) = 3000, *T* = 10. The sequence of cell areas *A* at each of the above Monte Carlo steps decrease as follows: *A* = 397, 364, 332, 299, 280, 250, 232, 219, 213, 208, and the sequence of cell perimeters first increases and then fluctuates: *P* = 74, 94.8, 98.2, 100.1, 99, 99.2, 99.1, 98.9, 99, 99.4.

A time sequence of cell shapes and accompanying forces is shown in Fig 3. At MCS step 1, the cell is far from its preferred configuration, and large forces are seen all along its edge. (Note that these forces are mostly directed outwards, with notable exceptions in non-convex regions of the boundary.) As the sequence of cell areas and perimeters evolves, (indicated in the caption), we find that the cell quickly obtains its target perimeter, and then the forces point inwards and the cell starts to shrink to obtain its target area. The irregular force directions and large magnitudes rapidly decline, so that by MCS 3, the force-field is more regular, and directed “inwards”. The cell becomes highly ramified, with thin protrusions so as to satisfy both area and perimeter constraints. After a few more MCS, starting around 7 MCS, the cell shape has equilibrated, and forces decrease to very low levels.

### Active forces from internal signaling

Several models have proposed signaling kinetics inside cells that result in forces of protrusion or retraction (powered by actin assembly or actomyosin contractility). There are many such models, at multiple levels of detail [13–16]. Among these is the simple “wave-pinning” model [17]. The model tracks the spatio-temporal distribution of a single GTPase in active and inactive forms, with interconversion, positive feedback to the activation rate, and distinct rates of diffusion of the two forms. We asked how internal signaling could be linked explicitly to forces on the cell edge in the CPM formalism. To address this question, we used the wave-pinning model as a prototype and benchmark (which is clearly replaceable by other signaling systems of interest, e.g. see [18, 19], and many other examples.) Accordingly, we set up a reaction-diffusion calculation in the interior of the CPM cell, as described in the Material and Methods to display the evolution of the internal GTPase activity field in parallel with the CPM force calculations. We assumed that a single signaling protein in two states (analogous to active and inactive forms of the GTPase RhoA) participates in reaction-diffusion kinetics inside the deforming “cell” and leads to edge contraction.

**Fig 4:**
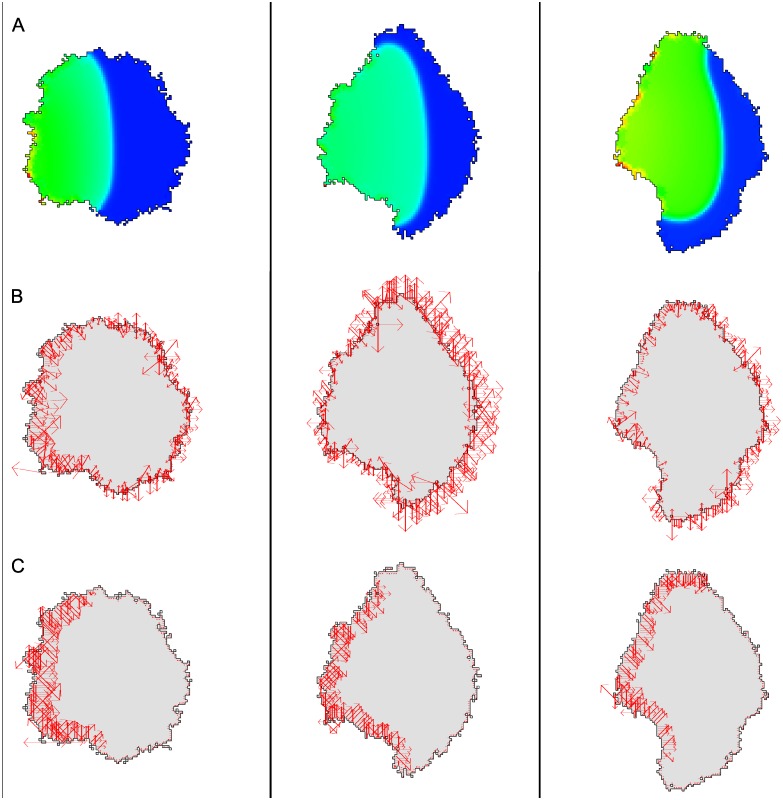
Active contractile forces from internal signaling. Shown is a time sequence (left to right, at 57, 107 and 157 MCS) of a moving cell whose shape changes in response to a polarizing internal signal (e.g. Rho GTPase). (A) The internal GTPase field (bright green at high values) based on the Wave-Pinning model. High levels of activity are assumed to create large local inwards contraction. (B) Total forces given by ∇*H* + *dH*_*u*_ along the perimeter of the deforming cell. (C) Forces due to the active contraction term (*dH*_*u*_). Forces are shown without smoothing or interpolation. Parameter values for CPM were: λ_*a*_ = 10, *a* = 4000, λ_*p*_ = 0, *J*(0, 1) = 5000, *T* = 50; parameter values for internal signaling: *β* = 40 (for *dH*_*u*_), the numerical redistribution radius was *r* = 3 (active rho), *r* = 75 (inactive rho). Parameters for internal reaction-diffusion system, and details for the numerical method are provided in the Supporting Information S1 File.

Regions of high Rho activity contiguous to the cell edge are shaded light green in Fig 4A. The internal chemistry leads to a force of protrusion, modeled by an additional term, Δ*H*_*u*_, superimposed on the Hamiltonian change. We assume Δ*H*_*u*_ = ±*βu*, where *β* > 0 is a constant, and *u* the local signaling activity level. We assume that the signal promotes contraction, so that Δ*H*_*u*_ is negative for retractions and positive for protrusion.

Results are shown in Fig 4A–4C as a time sequence of cell deformations from left to right. In Fig 4A, we see that chemical polarization is maintained, as described in previous studies [3, 13, 20, 21]. Contraction of the cell rear leads to the expansion of other cell edges based on the CPM area constraint. In Fig 4B and 4C, the total force field and the protrusive forces respectively are shown. Due to high signaling levels at the left edge of the cell, a contractile force pointing towards the right develops (Fig 4C). At the right side of the cell, forces due to the area and perimeter constraints point outwards. All in all, these forces result in migration of the cell to the right.

### Comparison with experimental force fields

Single cells can apply significant forces that remodel the extracellular matrix. In traction force microscopy, beads are embedded into a soft elastic substrate on which cells adhere. By tracking bead displacements, cell traction forces can be inferred. Such inverse methods quantify and reveal very detailed force fields. Traction forces are roughly aligned with the direction of the cell’s centroid, are highest in protruding regions and decline towards the cell’s centroid [22–26]. Via cell-cell adhesions, cells also apply forces on neighbouring cells and these forces can propagate through tissues [27].

**Fig 5:**
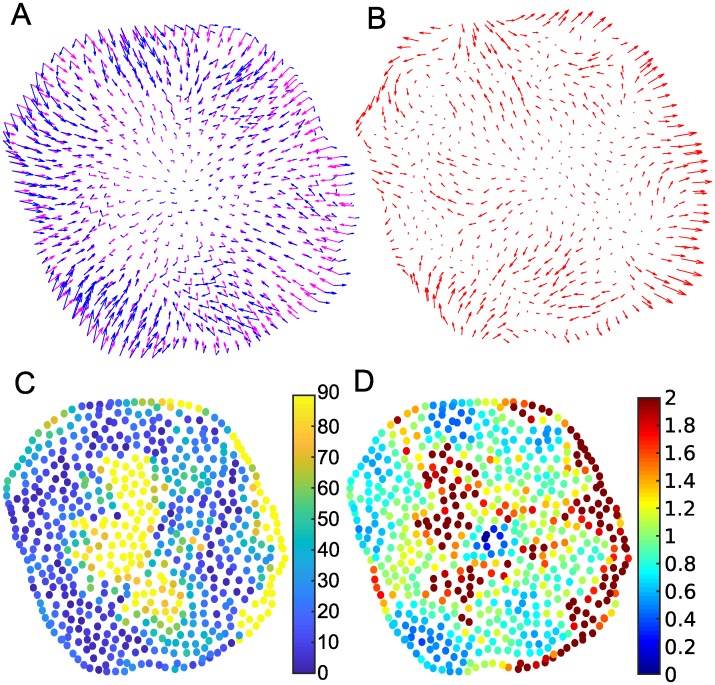
Comparing predicted forces to experimental data for a round cell. (A) Predicted CPM force fields (magenta arrows) and experimental data (blue arrows) (B) Difference of CPM force field and experimental force field (C) directional deviation (angle between predicted and experimentally observed force vectors), dark blue means forces align well. (D) relative magnitudes of the force fields, green means similar magnitude. Parameter values are given in Table S1 Table. See also S11 and S15 Figs.

**Fig 6:**
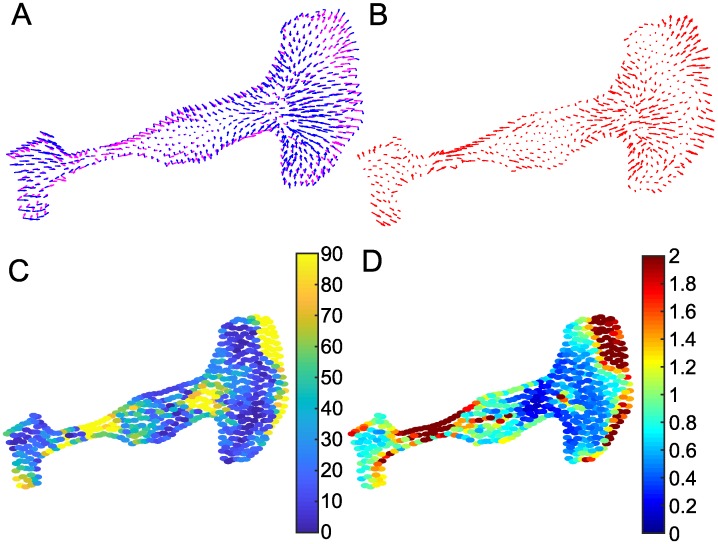
Comparing predicted forces to experimental data for a polarized cell. (A) Predicted CPM force fields (magenta arrows) and experimental data (blue arrows) (B) Difference of CPM force field and experimental force field (C) directional deviation (angle between predicted and experimentally observed force vectors), dark blue means forces align well. (D) relative magnitudes of the force fields, green means similar magnitude. Parameter values are given in Table S2 Table. See also S12, S13 and S16 Figs.

We asked how the predictions of the CPM-based force fields compare with data for actual traction forces observed in real cell experiments. Consequently, we utilized data kindly provided by the authors of [26] for two cancer cell lines. Several steps were needed to arrive at a shared grid, to select CPM parameters, and to compare magnitudes on a similar range, and adjust smoothing. Details are described in Methods and in the Supporting Information S1 File. Two examples are shown in Figs 5 and 6. Interestingly, our predicted force field looks quite similar to the force field predicted by a detailed rheological model of actin tension described in the same paper [26].

Figs 5A and 6A show observed (blue) and CPM predicted (magenta) force fields superimposed on the same grid. Overall, we find surprisingly good qualitative agreement, given the simplicity of the method. Experimental and predicted forces point roughly in the same direction for much of the cell shape. The concordance is particularly good for the round cell, where our approximation for centroid-pointing internal forces appears to be quite good. For the polarized cell in Fig 6, this agreement is less accurate, as two distinct “foci” appear to organize the force field in the experimental data. Figs 5B and 6B show the difference, *F*_*exp*_ − *F*_*CPM*_. As expected, there are regions in each cell where localized internal forces (not captured by CPM) result in significant deviation between experiments and predictions.

We compared directions of predicted and experimental forces at corresponding points. Results are shown in Figs 5C and 6C, with dark blue for points where observed and predicted forces are aligned, and yellow-orange for points at which the predicted direction deviates strongly from its observed value. Within a range of the cell edge, the model captures the direction of the forces reasonably well. This correspondence is quantified in S14(A), S14(C), S14(D), S15(A), S15(C), S15(D), S16(A), S16(C) and S16(D) Figs, showing an overall reasonable fit in terms of direction of forces. In the interior of the cell, force magnitudes are so small that directions carry large errors, and we cannot judge accuracy of the predictions. At the right side of both cells, predicted forces point inwards while observed forces point outwards. In the basic CPM Hamiltonian forces along the boundary can switch from pointing outwards to inwards only if the local curvature changes suddenly (see for instance Fig 2B and 2C), which does not happen in those regions in the two experimental cells.

We also compared relative force magnitudes, by plotting |*F*_*CPM*_|/|*F*_*exp*_| in Figs 5D and 6D. We find some regions of deviation, notably at the top right corner of the spindle-shaped cell. Figures S14B, S15B and S16B Figs, show overall deviation of the force magnitudes. CPM forces appear to be greater than the experimental forces. This stems from the fitting procedure: the linear interpolation in the interior is based on an assumption that forces decline towards the centroid. However, experimentally measured forces have local “hot spots” of large magnitude, so the fitting procedure adjusts the predicted CPM forces to be elevated overall, and, in particular, at the boundary of the cell.

We tested a variety of CPM parameter values, including those that provide optimal L2 norm fits of predictions to experimental data (See Tables S1 and S2 Tables). A comparison of results for distinct CPM parameters is shown in S11 and S12 Figs. The ‘optimal’ CPM parameter values vary over a much larger range for the round cell than for the polarized cell experimental data. There are many parameters λ_*a*_, λ_*p*_, *A*, *P* and *J*(0, 1) that determine the overall magnitude of the force, so it is not surprising that a good fit is obtainable with different values. (Interestingly, the target area in the top five parameter sets are all smaller than the experimental cell area, while the target perimeters are larger than the the actual experimental cell perimeters).

Finally, we also display a time series of cell movement in S13 Fig comparing the CPM force field with the experimental data for the polarized cell. During active cell motion, large traction forces are built up for translocation (long blue arrows) in the protrusive front of the cell in S13C and S13D Fig. The entire time series for the two cells can be viewed in S1 Movie (round cell) and S2 Movie (polarized cell). Throughout these evolving cell shapes, the predicted forces are reasonable. The direction of the forces only deviate from experimental forces in select regions (such as protruding fronts) and, as expected, regions of elevated forces are beyond the predictive power of the basic CPM. On one hand, the difference *F*_*exp*_ − *F*_*CPM*_ provides an estimate for spatially distributed active forces of protrusion/contraction in a motile cell. At the same time, such deviation suggests how to refine CPM models by inclusion of the cytoskeleton or adhesion distribution [13, 28], or by decomposing the single cell into subregions that represent focal adhesions or force-bearing internal structures.

### Interacting cells and adhesion forces

Forces between interacting cells are not easy to measure directly. However, they have been inferred from high-resolution traction-force measurements, for example by [29] using a force-balance principle and thin-plate FEM analysis.

Here, we asked whether our algorithm would predict intercellular forces in two or more cell that interact by adhesion. To investigate this question, we considered two scenarios, including simple adhesion and signaling-regulated motility in a pair of cells. Results are given below. Note that in the CPM, a high adhesive energy cost *J*, corresponds to low cell-cell adhesion. See [5, 30, 31] and references therein.

#### Varying adhesion strength

**Fig 7:**
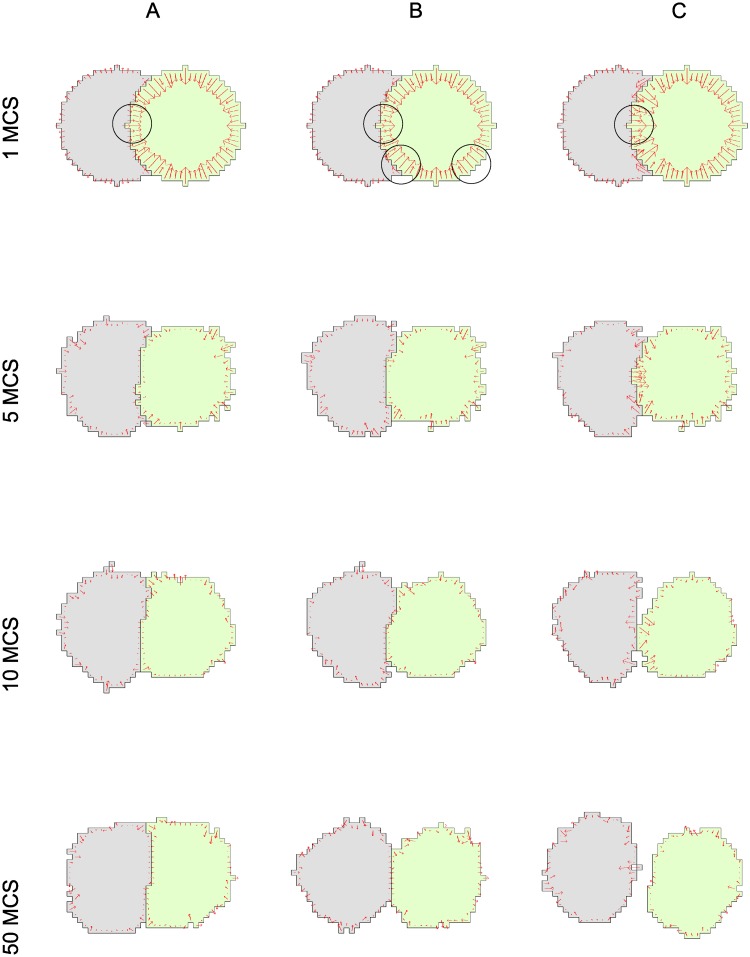
Forces due to cell-cell adhesion. Two CPM interacting cells in a time sequence from left to right. (A) cells adhere strongly *J*(1, 2) < 2*J*(0, 1), (B) neutral adhesion of cells to medium and to one another *J*(1, 2) = 2*J*(0, 1), (C) cells de-adhere, *J*(1, 2) > 2*J*(0, 1); CPM parameters used were λ_*p*_ = 0, λ_*a*_ = 8, *a* = 300, *J*(1, 0) = 1800, *T* = 300. We used *J*(1, 2) = 1800 (for the adhesive), 3600 (for the neutral) or 7200 (for the repulsive) cases.

For the adhesion experiment, we set λ_*p*_ = 0, to omit the perimeter constraint, and used only the target area and adhesive energy in the Hamiltonian. We explored several values of the adhesive energy *J*(1, 2) between cells, keeping both cells equally adherent to the ‘medium’, *J*(0, 1) = *J*(0, 2) = constant. Results are shown in Fig 7. Comparing forces at cell-cell interfaces for the three adhesive energies (centered black circles at 1MCS), we find that the force at a cell-cell interface is lowest in A, and highest in C. This is consistent with Eq (0.8). We find that highly ‘sticky’ cells (*J*(1, 2)< 2*J*(0, 1)) remain attached with a wide contact region, as shown in Fig 7A. For neutral cell-cell adhesion (*J*(1, 2) = 2*J*(0, 1)) in Fig 7B, the cells remain attached on a smaller contact interface. In this case, the round green cell initially (at 1 MCS), applies the same force magnitudes at every interface (note circled regions on the lower left and right of the green cell in Fig 7B). Finally, in Fig 7C, with *J*(1, 2) > 2*J*(0, 1), the energetically favored configuration is detached cells.

#### Two motile cells with internal signaling

**Fig 8:**
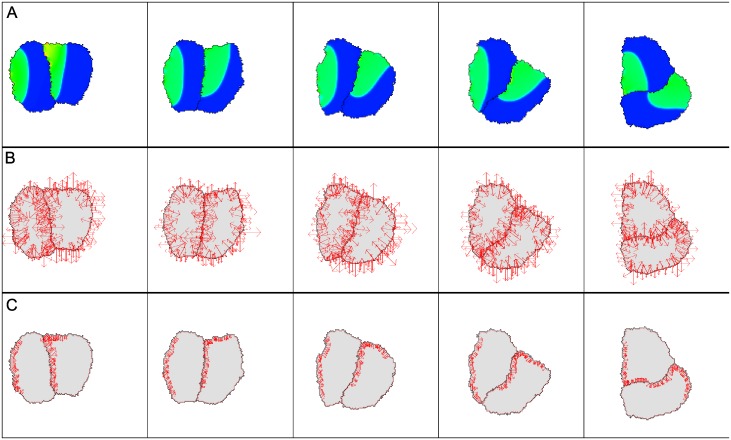
Edge forces in two adhering cells with internal signaling. (A) The level *u* of signaling activity (colors range from blue (low levels), to green, to red (high levels)), (B) total force exerted by each cell, (C) mutual forces due to signaling contraction alone. The cells polarize and circulate about one another. Parameters for the CPM are λ_*a*_ = 2, *a* = 2500, λ_*p*_ = 0, *J*(1, 0) = 30000 = *J*(1, 2), *T* = 200, *β* = 80 (for *dH*_*u*_). Parameters for the reaction-diffusion system are provided in the Supporting Information S1 File. High signaling activity (red) leads to local edge contraction. The configurations are shown at MCS 80, 180, 280, 380 and 480.

We next asked how internal signaling in each of two interacting CPM cells would affect their mutual adhesive forces. To explore this question, we assumed the wave-pinning signaling, as before, in each of the cells, starting initially with uniform signaling activities except for elevated activity along the left edge of each cell. Results are shown in Fig 8. The reaction-diffusion (WP) equations lead to rapid polarization of signal activity inside the cells, as before. High signal strength was associated with local contraction of the cell edge, and the area constraint then led to net motion. The two moving cells maintained contact due to their assumed high adhesion (low energy of cell-cell interfaces). While initially cells moved in roughly the same direction, at some later point, they started to rotate. This trend continued during the simulation. We show the internal signal distribution in Fig 8A, the total force computed from the CPM Hamiltonian in Fig 8B, and the active force due to the Rho-like signal in Fig 8C. It is apparent from the latter that forces cause a torque, leading to the observed rotation.

### Dynamic force fields in large multicellular aggregates

Finally, we sought to test our methods on simulations of larger cell aggregates. We asked whether the known dynamics of cell sorting, e.g. [1, 32–34], would correlate well with force fields that can now be directly visualized. For this purpose, we adopted the cell-sorting benchmark test cases, where dynamics are well-established. That is, we considered three typical cases, with two cell types and three distinct relative heterotypic and homotypic adhesions, leading to the classic checkerboard, separation, and engulfment scenarios.

**Fig 9:**
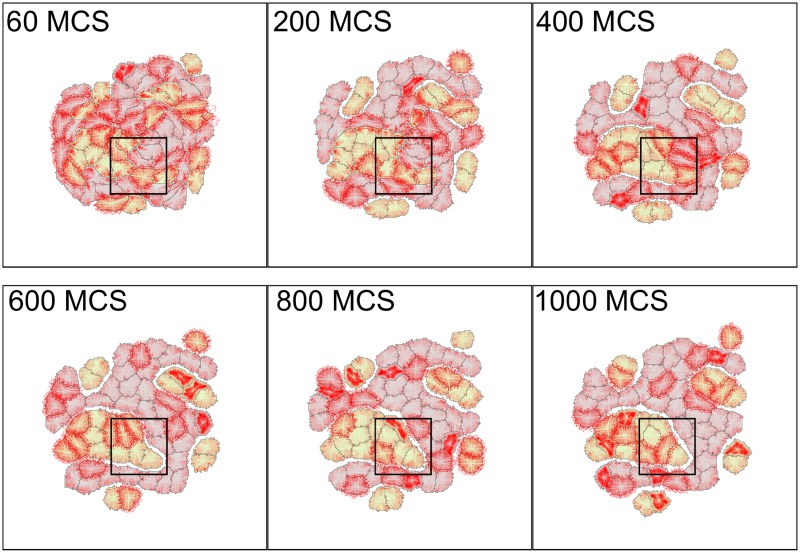
Separation cell sorting simulation with force visualization. Parameter values were *a* = 300, λ_*a*_ = 1000, *p* = 67, λ_*p*_ = 20, *J*(0, grey) = 1800, *J*(0, green) = 1800, *J*(grey, grey) = 900, *J*(green, green) = 900, *J*(grey, green) = 9000, *ξ*(*r*) = 18, and *r* = 3 for all neighborhood calculations. The cellular temperature *T* was set to 600. Zooms of the cells and forces inside the black squares can be viewed in S18 Fig. See also S19–S23 Figs.

Fig 9 shows a time sequence of the model cell aggregate for the “separation” case. Initially, cells are randomly mixed. Zooms of the cell configurations and forces inside the square regions can be seen in S18 Fig. Here, *J*(*AA*) = *J*(*BB*) = 900, *J*(*AB*) = 9000 (where A are green and B are grey cells), so that a relatively high energetic cost results from interfaces of unlike cell types (heterotypic interfaces). This means that the adhesive forces between green and grey cells are high and repulsive. Evident from Fig 9 are high forces that build up at heterotypic interfaces. (See zoomed regions shown in S18 Fig). By Monte-Carlo step 400, we find regions where cells have separated. Cell boundaries continue to adjust for some time, accounting for fluctuations between outwards and inwards-pointing forces in a given cell during these transients. By 1000 MCS, many of the separated boundaries have equilibrated to a large extent, and localized forces on those boundaries have relaxed. A few remaining cells are still compressed or stretched away from their preferred rest area and perimeter, and are seen to experience significant forces. Later, (5000 MCS, S19 Fig) separated clusters round up. Interestingly, these static images, in combination with the force-map allow for easy visualization of parts of the aggregate that are still actively deforming.

We show two related scenarios in the Supporting Information. A checkerboard cell-sorting case is illustrated in S20 and S21 Figs. The engulfment case is shown in S22 and S23 Figs.

## Discussion

Computational cell modelling promises to be a useful tool in testing hypotheses in single and collective cell behavior. A range of platforms is used, including force-based simulations of cells as self-propelled particles, points, spheres, or ellipsoids obeying Newtonian physics. A number of geometric cell models, including vertex-based (polygonal) cell simulations [4, 35] are based on energy minimization [36, 37] or on explicit springs and damping forces. One advantage of the Cellular Potts formalism, is that cell shape can be modelled in greater detail, is highly dynamic, and captures fluctuations seen in real cells.

Like any model, the CPM has its limitations, as described by [38]. Among these is the absence of an actual time scale in the “Monte Carlo Step”, mandating a definition of time scales by other methods (see, e.g., [5, 39, 40]). It is also argued that the CPM is based on phenomenological assumptions that may or may not be justified, or that it does not correspond to real biology. To some extent, this is true of any model of a cell, whether based on springs, solid objects, finite elements, or viscoelastic fluid. At the same time, the link between known biophysical properties of cells and CPM parameters has been firmly established by [5]. Here we have addressed a second common criticism of the CPM, namely that it bears no relationship to cell forces and mechanics. We have devised an explicit algorithm that links the Hamiltonian (a scalar energy) to a force-field that is consistent with that Hamiltonian.

Previous authors have combined classic CPM with external methods of tracking forces. Lemmon and Romer [25] assumed that a cell acts as a contractile unit resulting in a ‘first moment of area’ representation for the force distribution. Rens and Merks [7, 8] adopted this same method. Such a model produces reasonably realistic force fields, but are not necessarily consistent with the CPM Hamiltonian, as these forces are assigned independently of the assumed form of *H*. Albert and Schwarz [9] went in the opposite direction, devising a CPM Hamiltonian consistent with an arbitrary analytical expression for force on the cell edge. Their formula for the force was based on the curvature of the cell edge. (They approximate the curvature and normal direction along the pixellated cell edge to calculate force vectors. They then applied a smoother to distribute those forces in a region near the cell edge).

A brief mention of forces in simulations based on the CPM have previously appeared, e.g. in [10, 41, 42]. Some of these hint at the relationship to the CPM Hamiltonian. The most comprehensive and explicit of these is Magno et al. [5] who used the link between forces and gradients of potential energy ($\vec{F}=-\nabla H$) to write down the tension (*γ*), the pressure (Π), and the total force $\vec{F}$ for the basic CPM Hamiltonian,

$$\begin{array}{c} \gamma =\frac{\partial H}{\partial p}\;\Pi =-\frac{\partial H}{\partial a}\;\vec{F}=-\nabla H={\vec{F}}_{\Pi }+{\vec{F}}_{\gamma }=\Pi \nabla a-\gamma \nabla p. \end{array}$$

The authors used these relationships to derive a dynamical system for the size of a spherical cell, and to map cell size dynamics onto a 2-parameter plane with composite parameters. Our paper has taken motivation from their ideas to devise an algorithm for numerically computing forces directly from the CPM Hamiltonian for an arbitrary cell shape, and for multiple cells.

The group of Roeland Merks (Leiden U, formerly in part at Centrum Wiskunde & Informatica in Amsterdam) has longstanding efforts to link forces to the CPM Hamiltonian. Koen Schakenraad, a PhD student of Merks worked with one of us (EGR) and Merks on the idea of determining a Hamiltonian corresponding to forces postulated by Lemmon and Romer [25]. As the Lemmon and Romer forces are not necessarily gradient forces, finding such a Hamiltonian was not in general feasible. A second idea, also explored was to derive forces along the edge of a CPM cell from virtual work. A precursor to our paper is the thesis [43] of D.S. Laman Trip, also a student of Roeland Merks. In his simulations of tissue folding in that thesis, Trip displayed edge forces of CPM cells using a sum of virtual work done by spin flips in all directions at points on cell edges. The same idea of using virtual work is also mentioned in Appendix A of the PhD thesis [28] by one of us (EGR), though the thesis itself employed phenomenological calculations of cell forces other than those we propose. In contrast to the above, here we have chosen to connect CPM forces directly to finite difference approximations of the gradient of the Hamiltonian. While the above approaches are related, to our knowledge, our work is the first to provide a detailed algorithm that computes force components on the CPM cell edges, extends the field to the cell interior, reduces effects of the CPM grid by smoothing, and validates the predictions against experimental data.

While the CPM Hamiltonian predicts forces at cell edges, simple interpolation and smoothing to decrease grid effects were adopted. We showed that this approximation for the forces gives reasonable results for a range of cell shapes. Importantly, the most basic CPM Hamiltonian reproduces forces that are qualitatively consistent with experimental data and similar to detailed rheological models based on the same data [26]. Our algorithm applies not only to single cells but also to multicellular simulations. The computed force-fields provide insights to cell deformations accompanying three typical cell sorting experiments, where some but not all cells equilibrate with their neighbors. In such simulations, force fields within the clusters help track and understand the global and local dynamics of the cell collective. From the force-fields we can appreciate simulated cell motions and a more tangible connection between the Hamiltonian and cell behavior.

The approach is an approximation and has limitations that we summarize here. First, the classic Hamiltonian approximates a cell as an elastic element tending to retract/expand towards a specified rest area and rest-length circumference, which is a grossly simplified view of a cell. This feature is shared with other energy-based simulation platforms, e.g. [4, 35]. Moreover, in matching CPM predictions to experimental data, we find multiple sets of CPM parameters that give rise to very similar qualitative agreement. Improved calibration of the Hamiltonian to cells of given type would require more specific experimental data. These aims are beyond the scope of this paper.

A second issue is that the CPM Hamiltonian only changes for cell edge displacements, and so, only prescribes a force-field restricted to the cell edge pixels. We have assumed simple interpolation, with zero force at the cell centroid, but this is, to some extent, arbitrary. As seen in the experimental data in Fig 6, a polarized cell can have multiple points at which the force vanishes. Hence, the internal force field should not be over-interpreted. The basic Hamiltonian describes cell shape but not internal structures, so the derived force field is not appropriate for predicting localized regions of high internal forces. So, a second limitation is the attribution of forces to cell shape alone, neglecting active and heterogeneous structures (stress fibers, focal adhesions, cytoskeleton anisotropy, etc.). Some cell types (keratocytes, neutrophils) do not form focal adhesions, so experiments with these could be used to test the basic CPM predictions. Moreover, it is easy to extend the CPM to incorporate many more intracellular details. For example, in previous work, Mareé et al [13] assembled a more detailed internal signaling CPM model for a single motile cell that included actin filament orientation and pushing barbed ends (regulated by active Cdc42, and Rac), as well as edge contraction (due to GTPase Rho, as in our simple examples in Figs 4 and 8). Such details can be added for greater consistency with motile cells. Alternatively, incorporating other Hamiltonian terms such as directional polarity, or heterogenous, space-dependent, Hamiltonian terms, or representing a cell by a collection of CPM subdomains with distinct properties (“focal adhesions”, see also [12]) could be used to generalize these ideas. Each subdomain could have unique values of parameters λ_*a*_, λ_*p*_, *J* etc., leading to a refinement of the representation of a single cell by a collection of intracellular structures.

Another issue with CPM computations is that Monte Carlo steps are not scaled to actual time. This can be resolved by scaling the motion or cell cycle of CPM cells to real cell speeds or cycle times. Based on typical cell size, typical forces cells produce, and typical values of viscosity, one could also use the relationship *v* ≈ *F*/*ξ* to devise a time scale. (See also [39, 40].) Similarly, units of force could be assigned by calibrating the model against measurement of actual forces. In the data we obtained [26], all forces were nondimensionalized, which prevented an absolute force magnitude to be assigned.

We have carried out partial validation of the method against single-cell experimental data. Traction force microscopy has also been used to quantify patterns of stress in multicellular aggregates [29]. Stress is usually localized at the periphery of a cluster of cells, while at cell-cell interfaces the stress is lower [44], suggesting that the cluster acts as a single contractile unit. Inside the cluster, forces are highly dynamic and localized forces can occur due to cell proliferation or rotating motion [45]. Great progress has been made in visualizing force fields, and it is likely that modeling and computation will contribute to an understanding of how traction force are precisely regulated and what are consequences of the force dynamics on single and collective cell behavior.

## Materials and methods

### Cellular Potts Model

In the basic Cellular Potts model (CPM), each “cell” consists of a collection of connected lattice sites, assigned a unique index. Parts of the domain containing no cells are indexed 0 by convention. At each Monte-Carlo Step (MCS), and every edge pixel, the cell can either expand outwards by a pixel or retract inwards. (Formally, in the Ising terminology, “a spin flip copies the spin value of a source lattice site (${\vec{x}}_{s}$) to a target site (${\vec{x}}_{t}$)”.) This reconfiguration is typically carried out in a Moore neighborhood (one of eight nearest-neighbor pixels). The configuration change ($\sigma \left({\vec{x}}_{s}\right)\to \sigma \left({\vec{x}}_{t}\right)$) results in a change Δ*H*, in the Hamiltonian.

Our Hamiltonian is given by Eq (0.2) Many “spin flips” are attempted, but each is accepted with probability

$$\begin{array}{c} P\left(\Delta H\right)=\{\begin{array}{cc} 1 & \text{if}\;\Delta H+H_{0}<0\\ e^{-(\Delta H+H_{0})/T} & \text{if}\;\Delta H+H_{0}\geq 0. \end{array} \end{array}$$ (0.4)

where the “temperature” *T* ≥ 0 governs the magnitude of random fluctuations and *H*_0_, is a yield energy to be overcome. (Typically *H*_0_ = 0.) The CPM favors changes that decrease the energy of the configuration, while allowing fluctuations. We apply a connectivity constraint to avoid a cell fragmenting into two or more pieces.

We note that the CPM code used herein has been assembled, validated, and used by EGR, while a PhD student in the group of R. Merks (The Netherlands). That code has been refined over time to improve computation speed, number of cells that can be simulated, flexibility, and robustness. Nevertheless, we make no claims as to the relative merits of this CPM code. Other software platforms, such as CHASTE (U Oxford) or CompuCell3D (U Indiana, Bloomington) or any custom CPM code can be substituted for the one used in this paper.

### Approximating forces at points along cell boundaries

We discretize the gradient of the Hamiltonian, from Eq (0.3) as follows. Let *h* = Δ*x* = Δ*y* be the given grid size in 2D. For each point $\vec{x}$ on the border of a cell of configuration *σ*, consider a small local change, protrusion or retraction (Fig 1). The local “spin flip” at $\vec{x}$ produces a small change in the Hamiltonian. We can compute the force components $F_{x}\left(\vec{x}\right)$ and $F_{y}\left(\vec{x}\right)$ at $\vec{x}$ using a centered difference approximation to the first partial derivative (accurate to 2nd order):

$$\begin{array}{c} -F_{x}\left(\vec{x}\right)\approx \frac{\partial H}{\partial \sigma (\vec{x})}\cdot \frac{\partial \sigma (\vec{x})}{\partial x}\approx \frac{1}{2h}(H\left(\sigma +d_{x}\sigma \left(\vec{x}\right)\right)-H\left(\sigma -d_{x}\sigma \left(\vec{x}\right)\right)), \end{array}$$ (0.5)

and similarly for the component $-F_{y}\left(\vec{x}\right)$.

This algorithm defines forces at boundary points of every isolated cell. In the Supporting Information S1 File, we discuss other simplifications, refinements, and special cases. We implement steps to (1) improve accuracy and reduce grid effects (2) interpolate boundary forces to the cell interior, (3) generalize the idea to multiple cells and (4) compare predictions to measured force fields for real cells.

### Reducing the grid effects in perimeter calculations

As shown later in (0.8), it is known theoretically that forces associated with the Hamiltonian should be normal to the cell edge. Because the CPM approximates cell shape with pixels, the direction of forces from the above calculations before smoothing (S5 Fig) have a grid-effect. To obtain a better approximation of the normal vector and reduce this grid artifact, we employ smoothing using enhanced CPM neighborhood calculations inspired by [5]. Briefly, at each boundary site we define a weighted average of forces with weights given by “local cell perimeter” as computed using neighborhood summation. (We use a neighborhood radius *r* = 3 and *ξ*(*r*) = 18 [5] for rescaling the perimeter.) We find that this correction results in forces that are roughly orthogonal to the (refined) cell boundary. In the Supporting Information S1 File, we provide details and discuss how accuracy is affected by neighborhood radius. We also show that smoothing improves the agreement with the data (see also S10 Fig). For a discussion of additional effects, e.g. of grid direction and lattice anisotropy, see [46]. We have not corrected for such effects here.

### Phenomenological force fields in the interior

The methods described so far only provide a representation of the force field associated with the cell perimeter. We use simple interpolation from boundary sites to a point in the cell interior, typically the centroid of the region. This phenomenological choice, following [8, 9, 28], leads to a 2D force field.

### Intracellular reaction-diffusion system and protrusive forces

As a simple prototype to represent intracellular signaling that affects cell shape, we implement the wave-pinning reaction-diffusion (RD) model of [17] in the 2D cell interior, and compute the evolution of the RD system (with no-flux boundary conditions at the evolving cell boundary). Methods for our numerical computation, analogous to those of [13] are described in the Supporting Information S1 File. To link the internal chemical profile to forces on the cell boundary, we assume a Rho-like edge contractility: the “Rho activity”, *u*, close to the cell edge, is assumed to augment the local Hamiltonian changes by additional terms *dH* of the form ±*βu* for protrusions/retractions. In this way, the distribution of *u* can locally affect the probability of movement of the cell edge. After the cell edge moves, *u* is redistributed locally to avoid numerical mass loss, as described in the Supporting Information S1 File.

### Comparison with experimental data

We obtained traction force microscopy (TFM) data from Jocelyn Etienne and Claude Verdier for two cancer cell lines (T24 and RT112) as described by [26]. The authors plated cells on polyacrylamide gels containing fluorescent beads, and computed traction forces from bead displacements and known gel rheology [47]. We interpolated from their triangular to our rectangular grid (S8 Fig), and optimized the CPM parameters with respect to experimental data at one time point using a Latin Hypercube sampling method [48] (see Supporting Information S1 File and Tables S1 and S2 Tables). The CPM and experimental force fields are then displayed on the same grid, and their difference, directional deviation, and relative magnitudes are computed and displayed for comparative purposes.

### Generalization to multiple cells

For a system of multiple cells, we decompose the total Hamiltonian into contributions *H*^*i*^ made by each cell,

$$\begin{array}{c} H\left(\sigma \right)=\sum_{i=1}^{n}H_{A}^{i}+H_{P}^{i}+H_{J}^{i}\equiv \sum_{i=1}^{n}H^{i}, \end{array}$$ (0.6)

where $H_{A}^{i}$ and $H_{P}^{i}$ are as in Eq (0.2) for cell *i* and *H*_*J*_ is generalized to accommodate cell-cell adhesion energies,

$$\begin{array}{c} H_{J}^{i}=J\left(0,\tau \left(i\right)\right)P_{0i}+\frac{1}{2}\sum_{j=1}^{n}J\left(\tau \left(i\right),\tau \left(j\right)\right)P_{ij}. \end{array}$$ (0.7)

Here *n* is the number of cells, *τ*(*σ*) the cell type of cell *σ*, *P*_0*i*_ is the boundary length of cell *i* in contact with the medium and *P*_*ij*_ is the length of the cell *i*- cell *j* interface. (The factor $\frac{1}{2}$ corrects for double-counting of each interface.) The finite difference computation of forces along interfaces then follows from the single cell case. (See also the Supporting Information S1 File).

It has been shown elsewhere, e.g. [9], that force exerted by each cell can be reduced to the form

$$\begin{array}{c} {\vec{F}}_{i}\left(\vec{x}\right)=2\lambda \left(A-a\left(i\right)\right)\vec{n}+2\lambda_{p}\left(P-p\left(i\right)\right)\kappa \vec{n}+\kappa J\vec{n}, \end{array}$$ (0.8)

where *κ* is the curvature, $\vec{n}$ is the unit normal vector, and *J* is either *J*(0, 1) or *J*(*i*, *j*)/2.

## Supporting information

S1 File Details of methods. Technical details of calculations of CPM forces, smoothing, interpolation to cell interior, intracellular reaction-diffusion solver, comparison to experimental data, and multicellular force calculations.
(PDF)

S1 FigNeighborhoods for perimeter calculations.Neighborhoods $N(\vec{x},r)$ of various orders with radii *r* = 3, 5, 10 around a lattice site $\vec{x}$ (shown in red).(EPS)

S2 Fig Spin flips. Examples of four possible spin flips used to compute *F*_*x*_ based on our algorithm.
(EPS)

S3 Fig The effect of the neighborhood radius used for smoothing the cell boundary forces for ellipsoidal cells. (A) Sum of square errors (SSE) between normalized CPM force vectors and true unit normal vector (−*b* cos(*θ*), −*a* sin(*θ*)) to an ellipse with axes 10 and 20. The error is minimized at *r* = 14. (B) True normal vectors (green) to an ellipse with axis 10 and 20, compared to CPM forces smoothed with radius *r* = 3 (blue). (C) Neighborhood radii *r* corresponding to minimal SSE for ellipses with various axes lengths between 5 and 50. (D) Same as (B) but with smoothing radius *r* = 14.
(EPS)

S4 FigInterpolation used to compute force in cell interior.Interpolation is used to compute the force at a site $\vec{x}$ inside a CPM cell based on the centroid ${\vec{x}}_{C}$ and the force predicted by the CPM at a boundary site ${\vec{x}}_{M}$ along the ray connecting the centroid and the given site. The ray was determined by minimizing *α* in SI Eqn. (0.14).(EPS)

S5 Fig Comparison of interpolation methods. Magnitude of experimental forces vs the distance to the center of mass of the experimental cell. (A) round cell (B) polarized cell. We fitted a linear (red), quadratic (yellow) and exponential (purple) function to the data, obtaining similar lines.
(EPS)

S6 Fig Cell edge forces without smoothing. (A) A circular cell with an area of 401, perimeter of 74, and a diameter of 23. (B) An elliptical cell with an area of 629, perimeter 101, and short and long axis 21 and 41. (C) An irregular shape with area 301 and perimeter 118. (D) A highly irregular cell shape with area 400 and perimeter 146. Parameter values were *a* = 300, λ_*a*_ = 10, *p* = 100, λ_*p*_ = 10, *J*(0, 1) = 3000, *ξ*(*r*) = 18, and *r* = 3 for all neighborhood calculations. We used a grid of 50 by 50 lattice sites with Δ*x* = 1.
(EPS)

S7 Fig Cell edge forces with smoothing. As in S6 Fig but with smoothing applied to the boundary forces. The radius *r* = 3 was used for all neighborhood calculations.
(EPS)

S8 Fig Interior forces. Interior forces computed with no smoothing for the cell shapes shown in S6 Fig.
(EPS)

S9 Fig Mesh transformation from experimental data to CPM. Triangular mesh on which cell traction experimental data from [26] was supplied, and the corresponding CPM cell (spin value = 1).
(EPS)

S10 Fig Comparison of experimental data and CPM force predictions. Force fields from experimental data (blue) and CPM (magenta) using initial arbitrary CPM parameters for the round cell (A-B) and polarized cell (C-D). Radius of smoothing used was (A,B) *r* = 3, (C, D) *r* = 10. Regions of large deviation are circled.
(EPS)

S11 Fig Effect of fitted CPM parameters on agreement with experimental data (round cell). Fitting CPM parameters: Experimental data (blue) and CPM (magenta) force fields for the round cell using the second (A), third (B), fourth (C) and fifth (D) best CPM parameter values. Parameter values are given in S1 Table.
(EPS)

S12 Fig Effect of fitted CPM parameters on agreement with experimental data (polarized cell). As in S10 Fig but for the polarized cell using the second (A), third (B), fourth (C) and fifth (D) best CPM parameter values in S2 Table.
(EPS)

S13 Fig Forces computed over time during cell motion. A time sequence of cell motion and force fields from [26] showing experimental data (blue) and CPM (magenta) force fields. The CPM parameters were as in S11 Fig and row 1 of S2 Table.
(EPS)

S14 Fig Comparison of directions and magnitudes of forces from experimental data and from CPM predictions. Correspondence between experimental data and CPM predicted forces. Boxplots showing distributions of (A) the directional deviation (angle between experimental and model forces), (B) relative magnitudes of forces (C) deviation of *x* components and (D) *y* components of the forces.
(EPS)

S15 Fig Scatter-plots comparing experimental and CPM predicted forces for the round cell. (A) angle of the force, (B) magnitude of the force, (C) *x* component of the force, (D) *y* component of the force.
(EPS)

S16 Fig Scatter-plots comparing experimental and CPM predicted forces for the polarized cell. As in S15 Fig but for the polarized cell.
(EPS)

S17 Fig Force calculations for multiple cells. Spin-flips used to approximate the force exerted by the grey cell at cell-cell interfaces (A) CPM spin-flip modeling extension of the grey cell, shifting the cell-cell interface to the right (B) CPM spin-flip modeling a retraction of the grey cell, shifting the cell-cell interface to the left.
(EPS)

S18 Fig Zooms of the separation cell-sorting simulation. A magnification of the square regions in Fig 9 of the main text. Parameter values were *a* = 300, λ_*a*_ = 1000, *p* = 67, λ_*p*_ = 20, *J*(0, grey) = 1800, *J*(0, green) = 1800, *J*(grey, grey) = 900, *J*(green, green) = 900, *J*(grey, green) = 9000, *ξ*(*r*) = 18, and *r* = 3 for all neighborhood calculations. The cellular temperature *T* was set to 600.
(PNG)

S19 Fig A separation cell-sorting simulation at 5000 MCS. Parameter values were *a* = 300, λ_*a*_ = 1000, *p* = 67, λ_*p*_ = 20, *J*(0, grey) = *J*(0, green) = 1800, *J*(grey, grey) = *J*(green, green) = 900, *J*(grey, green) = 9000, *ξ*(*r*) = 18, and *r* = 3 for all neighborhood calculations. The cellular temperature *T* was set to 600. Some cells are still experiencing large forces since the cluster is still not equilibrated.
(PNG)

S20 Fig A checkerboard cell-sorting simulation. Parameter values were as in S19 Fig but with *J*(grey, grey) = *J*(green, green) = 7200, *J*(grey, green) = 1800.
(PNG)

S21 Fig A checkerboard cell-sorting simulation at 5000 MCS. Parameter values were as in S20 Fig.
(PNG)

S22 Fig Engulfment cell-sorting simulation. Parameter values were were as in S19 Fig but with *J*(0, grey) = 1800, *J*(0, green) = 9000, *J*(grey, grey) = 1800, *J*(green, green) = 1800, *J*(grey, green) = 3600.
(PNG)

S23 Fig Engulfment cell-sorting simulation at 5000 MCS. Parameter values as in S22 Fig.
(PNG)

S1 Table CPM parameter fits for round cell. Top 5 parameters sets from the Latin hypercube sampling for the round cell, all giving very similar fits. The first set is used in the main text and the force fields for 2-5 are given in S10 Fig.
(PDF)

S2 Table CPM parameter fits for polarized cell. As in Table S1 Table, but for the polarized cell. Multiple parameter sets give very similar fits with SSE around 1.3e6. First set is used in the main text and the force fields for 2-5 are given in S11 Fig.
(PDF)

S1 Movie Forces computed over time for motion of the round cell. A time sequence of cell motion and force fields from [26] showing experimental data (blue) and CPM (magenta) force fields. The CPM parameters were as in S11 Fig and row 1 of S2 Table.
(AVI)

S2 Movie Forces computed over time for motion of the polarized cell. As in S1 Movie, but for the polarized cell.
(AVI)
