# Supplementary material for: From energy to cellular forces in the Cellular Potts Model: An algorithmic approach
Source: PLoS Comput Biol. 2019 Dec 11;15(12):e1007459. doi: 10.1371/journal.pcbi.1007459 (PMC6927661; doi:10.1371/journal.pcbi.1007459)
Supplement: S1 File — Technical details of calculations of CPM forces, smoothing, interpolation to cell interior, intracellular reaction-diffusion solver, comparison to experimental data, and multicellular force calculations. (PDF) [file pcbi.1007459.s001.pdf]

Supporting Information for: From Energy to Cellular Force in the Cellular Potts Model

Elisabeth G. Rens[1,2], Leah Edelstein-Keshet[1,*]

1 Department of Mathematics, University of British Columbia, Vancouver, BC, Canada, V6T 1Z2, **2** rens@math.ubc.ca, * Corresponding author: keshet@math.ubc.ca

Details of Methods.

Cellular Potts Model (CPM)

In the CPM, cell shape is described on a discrete lattice and evolves through minimization of a Hamiltonian, analogous to an energy. Dynamic changes in cell shapes, interfaces, and positions result in changes in the Hamiltonian. The dynamics of the system are governed by the general principle of energy minimization, allowing for fluctuations that are akin to "thermal noise". The latter helps to avoid getting trapped in local energy minima. The Hamiltonian is minimized by a Metropolis type algorithm, where cell edge movements are iteratively attempted and actually carried out if the movement decreases the Hamiltonian. For surveys of CPM and its applications, see [1, 2].

In the CPM, both shapes and positions of each "cell" (configuration denoted by σ) evolve in time. Changes that minimize the Hamiltonian are favored. At each simulation step (Monte Carlo Step, MCS) every boundary pixel of each cell may "protrude" or "retract". (Formally, these changes are denoted "spin-flips", each corresponding to copying the spin value of a source lattice site ($\vec{x}_s$: source site) onto a neighboring target lattice site ($\vec{x}_t$: target site; $\sigma(\vec{x}_s) \to \sigma(\vec{x}_t)$). The target sites are typically the eight nearest-neighbor pixels of the source site (Moore neighborhood). While many such small spin-flips are tested, those that are actuated depend on the resulting changes in the Hamiltonian (ΔH) as described in the Main Text.

A common issue raised in the literature is that CPM simulations are not in correspondence with Newtonian forces, and hence non-physical. However, as argued eloquently in [2], the energy-based CPM formalism suggests direct correspondence with a force representation, and we utilize such ideas below.

Relating forces to the Hamiltonian

The force is related to the Hamiltonian (energy) by

$$\vec{F} = -\nabla H = -\left(\frac{\partial H}{\partial x}, \frac{\partial H}{\partial y}\right), \quad \nabla = \left(\frac{\partial}{\partial x}, \frac{\partial}{\partial y}\right).$$

For the Hamiltonian given in the main text, it has been noted by e.g., [3] that the same force can also be expressed in the form

$$\vec{F}(\vec{x}) = 2\lambda(A-a)\vec{n} + 2\lambda_p(P-p)\kappa\vec{n} + J(0,1)\kappa\vec{n}, \tag{0.1}$$

where, $\vec{n}$ is the inward unit normal vector at the cell boundary and κ is the local curvature.

Approximating forces at points along cell boundaries

For $h = \Delta x = \Delta y$ the grid size and $\vec{x}$ a point of a cell boundary, let σ be the cell configuration. Then a single "spin flip" at $\vec{x}$ produces a small change in the Hamiltonian. This idea allows us to approximate $F_x = -\partial H/\partial x \approx -\Delta_x H/\Delta x$, and similarly for F_y.

CPM spin-flips are variations of the cell configuration, σ. We define $d_x\sigma, d_y\sigma$ as spin-flips in the x and y directions that displace the cell boundary to the left or right relative to the given lattice site.

The centered difference approximation to the first partial derivative results in

$$-F_x(\vec{x}) \approx \frac{\partial H}{\partial \sigma(\vec{x})} \cdot \frac{\partial \sigma(\vec{x})}{\partial x} \approx \frac{1}{2h} \left(H(\sigma + d_x\sigma(\vec{x})) - H(\sigma - d_x\sigma(\vec{x})) \right), \quad (0.2)$$

and similarly for the component $-F_y$. Spin flips along any flat edge do not change the configuration. Hence the direction of the force at such points would always be normal to the flat edge.

Using the fact that $H(\sigma + d\sigma) = H(\sigma) + dH(\sigma \to \sigma + d\sigma)$, we can rewrite the above as

$$-F_x(\vec{x}) \approx \frac{1}{2h} \left(\Delta H(\sigma \to \sigma + d_x\sigma(\vec{x})) - \Delta H(\sigma \to \sigma - d_x\sigma(\vec{x})) \right). \quad (0.3)$$

There are some special cases. If a site $\vec{x}$ is positioned directly between two boundary points, as shown in S2 Fig, then there are four possible spin-flips that affect the configuration at $\vec{x}$: shifting the left-most cell edge out/in, or shifting the right-most edge out/in. These lead, respectively to the two approximations

$$-F_x(\vec{x}) \approx -F_x(\vec{x})^{\text{left}} = \frac{1}{2h} \left(H(\sigma + d_x^{\text{left}}\,\sigma(\vec{x})) - H(\sigma - d_x^{\text{left}}\,\sigma(\vec{x})) \right), \quad (0.4)$$

or

$$-F_x(\vec{x}) \approx -F_x(\vec{x})^{\text{right}} = \frac{1}{2h} \left(H(\sigma + d_x^{\text{right}}\,\sigma(\vec{x})) - H(\sigma - d_x^{\text{right}}\,\sigma(\vec{x})) \right). \quad (0.5)$$

To avoid a bias in a particular direction, we resolve this by taking the average of Eqs. 0.4 and 0.5, so that

$$-F_x(\vec{x}) \approx -\frac{1}{2} \left(F_x(\vec{x})^{\text{left}} + F_x(\vec{x})^{\text{right}} \right). \quad (0.6)$$

This approach is illustrated in S2 Fig. Similar computations apply to F_y, as before.

Reducing the grid effects in perimeter calculations

Pixellation introduces artifacts in the perimeter of a cell. Approximating the cell perimeter as the sum of lattice edges (or number of lattice sites along the edge) is quite poor [2], introducing a large grid effect. We adopt the correction by [2] with the following neighborhood calculation:

$$P \approx \frac{1}{\xi(r)} \sum_{\vec{x}:\sigma(\vec{x})=1} \; \sum_{\vec{x}\prime \in N(\vec{x},r)} 1 - \delta(\sigma(\vec{x}), \sigma(\vec{x}\prime)). \quad (0.7)$$

Here $N(\vec{x}, r)$ is the collection of neighboring sites of $\vec{x}$ within a range r and $\xi(r)$ is a scaling factor. (See S1 Fig.) This summation counts the number of neighboring sites of $\vec{x}$ that are outside of the cell, i.e., how much $\vec{x}$ contributes to the perimeter. (Note that $\sigma : \Lambda \to \mathbb{N}$ is the cell index or "spin value", so that the delta function is nonzero only if $\vec{x}, \vec{x}$ are both sites inside the same cell.)

A Moore neighborhood is often chosen (S1 Fig) to compute the perimeter term (or adhesive energy term) in the Hamiltonian. For a neighborhoods with radius r,

$$N(\vec{x}, r) = \{\vec{x}\prime : ||\vec{x} - \vec{x}\prime|| \leq r\}. \tag{0.8}$$

The larger the radius, the better the approximation of the perimeter (provided that the radius is not too large relative to cell size) [2]. Finally, the summation is normalized by the scaled factor ξ that corrects for the neighborhood radius [2]. We typically use a radius of 3 pixels for the neighborhood.

Smoothing the forces

We refine the direction of the forces on the cell edge as follows. First we define $\vec{N}$ to be a weighted average of forces within a neighborhood as

$$\vec{N}(\vec{x}) = \sum_{\vec{x}\prime \in N(\vec{x}, r)} \delta(\sigma(\vec{x}), \sigma(\vec{x}\prime)) w(\vec{x}\prime) \vec{F}(\vec{x}\prime), \tag{0.9}$$

where

$$w(\vec{x}\prime) = \sum_{\vec{y} \in N(\vec{x}\prime, r)} 1 - \delta(\sigma(\vec{y}), \sigma(\vec{x}\prime)). \tag{0.10}$$

These weights count the number of neighborhood sites of $\vec{x}\prime$ outside of the cell, i.e. the contribution of $\vec{x}\prime$ to the perimeter. We scale the vector $\vec{N}$ to obtain a unit vector $\vec{n}$, and then define the refined force as

$$\vec{F}'(\vec{x}) = |\vec{F}(\vec{x})| \vec{n}(\vec{x}), \quad \text{where} \quad \vec{n} \approx \frac{\vec{N}}{|\vec{N}|}. \tag{0.11}$$

Here $|\vec{F}|$ is the magnitude of the force given by our finite difference approximation. In Eq. 0.10, we use a neighborhood radius of $r = 3$, as recommended by [2].

Optimal neighborhood size for smoothing

We numerically investigated the relationship between the smoothing neighborhood radius r, used in Eq. 0.9, and the accuracy of the smoothed force vector. To do so, we took an elliptical shape, as in S3 Fig, for which the boundary normal vector is known:

$$\frac{x^2}{a^2} + \frac{y^2}{b^2} \leq, \quad \vec{n}_{\text{ellipse}} = (-b\cos(\theta), -a\sin(\theta)). \tag{0.12}$$

We first created a pixelated ellipse and displayed the approximated normal vectors for $r = 3$ in blue compared to the actual normal vectors of (0.12) (green). We next compared results of the smoothing algorithm of (0.9) with various values of the radius r. In each case, we compute the L^2 norm (sum of squared errors, SSE),

$$\text{SSE} = \sum_{\text{membrane sites}} ||f_{\text{cpm}} - f_{\text{ellipse}}||^2,$$

between the approximate (smoothed) normal direction and $\vec{n}_{\text{ellipse}}$. S3 FigA shows the SSE for an ellipse with axes $a = 10$ and $b = 20$ as a function of the radius r. We find that the optimal radius is $r = 14$. Panel D shows the smoothed normal vectors for $r = 14$ (blue) compared to actual normal vectors (green), showing that, indeed, there is improvement over a smoothing radius of $r = 3$.

We asked how the ellipse aspect ratio affects this conclusion. To test this, we varied the axes a and b of the ellipse, each from 7.5 to 47.5 in steps of 2.5. For each of these

ellipses we computed the optimal r as before. Results are shown in S3 FigC, where the elliptical axis a is on the x-axis and various values of b are shown with different colors. The optimal smoothing radius r increases roughly linearly with the length of either elliptical axis. Importantly, the SSE increases dramatically as r becomes too large relative to the cell size (Panel D), implying that larger r values are to be avoided. If cell shape is irregular, with small structures to be resolved, then large r values are inappropriate. We adopted $r = 3$ as a compromise.

Phenomenological force fields in the interior

The centroid of the cell shape is

$$\vec{x}_c = (< x >, < y >), \tag{0.13}$$

where $< \cdot, \cdot >$ denotes an average over all sites inside the cell. The goal is to define a force vector at every interior point $\vec{x}$ inside the cell. We do this by interpolating force vectors from the cell boundary to the centroid along straight line rays, assuming that net force at the centroid vanishes.

At each internal site $\vec{x}$ we identify the boundary site $\vec{x}_m$ on such a ray to the centroid,

$$\vec{x}_m = \underset{\vec{x}_m \in \mathbb{B} \wedge |\vec{x}_m - \vec{x}_c| > |\vec{x} - \vec{x}_c|}{\operatorname{argmin}} \alpha(\vec{x} - \vec{x}_c, \vec{x}_m - \vec{x}_c), \tag{0.14}$$

where $\mathbb{B}$ is the set of membrane sites and α denotes the angle between the given vectors. (See S4 Fig.)

We asked what type of interpolation would be appropriate. To decide on a trend to adopt, we used the experimental data to plot the magnitude of forces vs the distance to the centre of mass of the cell (S5 Fig). For the round cell, the trend looks roughly linear, whereas the polarized cell the trend is less clear, as expected. (In particular, the polarized cell appears to have more than one point where forces vanish.) We compared a linear, quadratic and exponential fit to the data. All three trends appear to fit similarly (S5 Fig). Consequently, we choose a simple linear interpolation.

Using the linear interpolation, the force at $\vec{x}$ is assigned to be

$$\vec{F}(\vec{x}) = \vec{F}(\vec{x}_m) - \frac{|\vec{x} - \vec{x}_m|}{|\vec{x}_c - \vec{x}_m|}\vec{F}(\vec{x}_m). \tag{0.15}$$

To smooth the vector field, we take an average over all boundary pixels that are neighbours to $\vec{x}_m$:

$$\vec{F}(\vec{x}) = \left\langle \vec{F}(\vec{x}\prime) - \frac{|\vec{x} - \vec{x}\prime|}{|\vec{x}_c - \vec{x}\prime|}\vec{F}(\vec{x}\prime) \right\rangle_{\vec{x}\prime \in N(\vec{x}_m, r) \wedge \vec{x}\prime \in \mathbb{B}_i}. \tag{0.16}$$

Intracellular reaction-diffusion system and protrusive forces

For the internal signaling simulations, we used the wave-pinning reaction-diffusion model of [4] as a simple test of the method. Here $u(x,t), v(x,t)$ are active and inactive forms of a signaling protein, e.g. Rho GTPase, satisfying the reaction-diffusion equations,

$$\frac{\partial u}{\partial t} = D_u \nabla^2 u + f(u,v), \tag{0.17a}$$

$$\frac{\partial v}{\partial t} = D_v \nabla^2 v - f(u,v), \tag{0.17b}$$

$$f(u,v) = \left(k + \gamma \frac{u^2}{u_0^2 + u^2}\right) v - \eta u. \tag{0.17c}$$

Parameters for the reaction-diffusion system were as follows: $D_u = 0.04$, $D_v = 1$, $\eta = 5.2$, $k = 1$, $\gamma = 30$. Initial conditions were $u = 0.04488, v = 0.4$, with an elevated activity region with $u = 4$ along the left edge of the cell. The reaction-diffusion equations are simulated in the irregular domain of the CPM cell, with 2000 iterations of the RD system per MCS. The spatial and time discretizations were $dx = 0.015, dt = 0.00005$. This leads to 0.1s per MCS [8].

We then assign a Hamiltonian difference dH to sites along the cell edge following the rule

$$dH_u = \begin{cases} -\beta\, u(\text{target site}) & \text{for cell retractions,} \\ +\beta\, u(\text{source site}) & \text{for cell extensions.} \end{cases}$$

During each MCS, we update the chemical signaling field in the cell interior 1000 times. After an edge extension, we locally adjust the chemical distribution to avoid artifacts of numerical mass loss as follows: find all sites x within a range r of the source site s, let $T_s(u) = \Sigma_r u$ be the total amounts of u, within that range around the source site; define a scaling factor

$$f = \frac{T_s(u)}{T_s(u) + u(s)}.$$

Set the level of chemical activity in the new site (target site t) to $u(t) = u(s) \cdot f$; at every one of the surrounding sites x, rescale $u(x) \cdot f$. This ensures that the chemical level of the source site is copied into the target site, but the total level of signaling activity does not change. After an edge retraction, we carry out a similar redistribution around the target site t, and scaling, but using the scaling factor

$$f = 1 + \frac{u(s)}{T_t(u)}.$$

The level of active chemical u is then locally scaled by this factor. If $T_t(u) = 0$ (i.e all neighbouring sites did not contain GTPase), we redistribute by adding GTPase: $u(x) = u(x) + (u(s)/\#\text{neighbours})$.

For the inactive form v we do the same as above. We used different neighborhood balls for redistributing active versus inactive signaling levels, as the ranges of diffusion of these differ. Since $D_v = 25D_u$, the range r is selected as $r_v = 25r_u$. Hence, the radii for redistribution were 3 for u, and 75 for v.

Comparison to experimental data

The data for cell shape and traction force from [5] was provided to us on a triangular mesh. We first converted to the CPM square grid. Using the MatLab function "imresize", the data was reduced to an 80×60 grid. Cell shapes were extracted by thresholding (Grey-scale values > 0.5 set to 1, < 0.5 set to 0, see S9 Fig. Coordinates were scaled to $-1 \leq x, y, \leq 1$ in both data and CPM, and then images were superimposed. We identified the CPM coordinate closest to each data point and compared forces (CPM vs data) at these corresponding points.

The appropriate CPM parameters $(\lambda, \lambda_p, J(0,1), A, P)$ are not known a priori for the given cell types and conditions. These parameters are assigned as follows. First, we determined area and perimeter of the (scaled) data cells; these were found to be $a = 1873, p = 163$ (round cell) and $a = 949, p = 218$ (polarized cell). We then choose smaller target CPM area and perimeter for the given cell. Next, we select initial values for $\lambda, \lambda_p, J(0,1)$ such that all three corresponding terms in the Hamiltonian have a roughly similar contributions (λ=0.1, λ_p=1, $J(0,1)$=250, A=500, P=50.)

It was not immediately clear what the best value of the smoothing radius, r, is for smoothing the edge forces. Hence, we first tested $r = 3$, as for our original method. Results are shown in S10 Fig. Because the direction of forces at protruding regions

(circled) deviated strongly, we adopted a smoothing radius of $r = 10$. We rescaled the CPM parameters $\lambda, \lambda_p, J(0,1)$ by a constant scale factor α by minimizing the L^2 norm between scaled CPM forces and data forces. This brings the force magnitudes to a common scale. We found that $\alpha_{\text{round}} = 0.5907$ for the round cell and $\alpha_{\text{polarized}} = 0.2241$ for the polarized cell.

The above values of CPM parameters resulted in favorable comparisons between CPM forces and experimental data forces. However, we investigated whether a different CPM parameter set would lead to a better fit. To do so, we defined a range of parameter values: $0 < \lambda_a < 1 \cdot \alpha$, $0 < \lambda_p < 5 \cdot \alpha$, $0 < J(0,1) < 2000 \cdot \alpha$, $0 < A < 3000$, $0 < P < 300$, $0 < r < 50$ (for membrane force smoothing). These ranges were binned into 10000 bins. Eventually, we included r for edge smoothing into our fitting procedure, to get a better idea of what r should be given a certain cell size and eccentricity (see also S3 Fig). Note that sampling leads to non-integer values of r. We used a Latin Hypercube sampling [9] (in MatLab), and sampled 100000 times. For each sample, we calculate the L2 norm between CPM and data forces. Overall, we found that different parameter sets gave very similar results. We used the first set in the Tables S1 Table and S2 Table for figures in the main text. In S14 Fig to S16 Fig we in detail compare predicted vs experimental forces.

In general, CPM parameters would, ideally, be optimized for a given cell type and conditions, and then used for predicting and validating other data not used in such optimization. Our data was limited, and so this optimization was beyond the scope of this initial series of tests.

Multiple cells and forces on cell-cell interfaces

For multicellular aggregate, we decompose the total Hamiltonian into contributions H^i made by each cell.

$$\begin{aligned} H(\sigma(\Lambda)) &= \sum_{i=1}^{n} \left(\lambda_a (A(i) - a)^2 + \lambda_p (P(i) - p)^2 + J(0, \tau(i)) P_{0i} + \frac{1}{2} \sum_{j=1}^{n} J(\tau(i), \tau(j)) P_{ij} \right) \\ &= \sum_{i=1}^{n} H_A^i + H_P^i + H_J^i = \sum_{i=1}^{n} H^i, \end{aligned} \tag{0.18}$$

where P_{0i} is the length of the membrane of cell i that is in contact with the medium:

$$P_{0i} = \frac{1}{\xi(r)} \sum_{\vec{x}:\sigma(\vec{x})=i} \sum_{\vec{x}\prime \in N(\vec{x},r) \wedge \sigma(\vec{x}\prime)=0} 1, \tag{0.19}$$

and P_{ij} is the length of the interface between cell i and cell j:

$$P_{ij} = \frac{1}{\xi(r)} \sum_{\vec{x}:\sigma(\vec{x})=i} \sum_{\vec{x}\prime \in N(\vec{x},r) \wedge \sigma(\vec{x}\prime)=j} 1. \tag{0.20}$$

We use CPM spin-flips to calculate the force due to shifting the cell-cell interface (see S17 Fig).

For a multicellular cluster at equilibrium, the force-balance principle states that traction forces must integrate to zero over the cell (or cell-cluster) footprint in 2D [6, 7]. This can serve as an additional check on the predictions.

Other Simulations of Multicellular aggregates

We start with a checkerboard type simulation as shown in S19 Fig. Here, the heterotypic adhesive forces are higher than the homotypic adhesive forces, so that cells of different type repel each other resulting in a checkerboard pattern. We provide zooms of group of grey cells within the clusters. The forces between grey cells are high and repulsive. As time proceeds, this allows those cells to move away from each other and the green cells to push in between them. In the whole cluster, we observe a decrease in forces, indicating that the aggregate is going towards a force balance. The pattern stabilizes somewhat (see configuration at 5000 MCS in S20 Fig) but due to random fluctuations and pressure on the cells in the interior of the cluster, high forces appear and disappear at different spots in the clusters.

If the adhesive forces between green cells and the medium is very high, the grey cells will engulf the green cells. An example engulfment simulation is given in S21 Fig. Here the zoomed views track a region around the boundary of the cluster. Since green cells have high forces with the medium, they move into the clusters to avoid contact with the surrounding medium. The engulfment is not completed within the time frame shown here, but after 5000 MCS (S22 Fig) the engulfment is more or less complete.

List of Supporting Information Figures

(For captions see main text)

S1 Fig. Neighborhoods for perimeter calculations.

S2 Fig. Spin flips.

S3 Fig. The effect of the neighborhood radius used for smoothing the cell boundary forces for ellipsoidal cells.

S4 Fig. Interpolation used to compute force in cell interior.

S5 Fig. Comparison of interpolation methods.

S6 Fig. Cell edge forces without smoothing.

S7 Fig. Cell edge forces with smoothing.

S8 Fig. Interior forces.

S9 Fig. Mesh transformation from data to CPM.

S10 Fig. Comparison of data and CPM force predictions.

S11 Fig. Effect of fitted CPM parameters on agreement with experimental data (round cell).

S12 Fig. Comparison of data and CPM force predictions (polarized cell).

S13 Fig. Forces computed over time during cell motion.

S14 Fig. Comparison of directions and magnitudes of forces from data and from CPM predictions.

S15 Fig. Scatter-plots comparing experimental and CPM predicted forces for the round cell.

S16 Fig. Scatter-plots comparing experimental and CPM predicted forces for the polarized cell.

S17 Fig. Force calculations for multiple cells.

S18 Fig. A separation cell-sorting simulation.

S19 Fig. A checkerboard cell-sorting simulation.

S20 Fig. A checkerboard cell-sorting simulation at 5000 MCS.

S21 Fig. Engulfment cell-sorting simulation.

S22 Fig. Engulfment cell-sorting simulation at 5000 MCS.

S1 Table. CPM parameter fits for round cell

S2 Table. CPM parameter fits for polarized cell

References

1. Marée AF, Grieneisen VA, Hogeweg P. The Cellular Potts Model and biophysical properties of cells, tissues and morphogenesis. In: Anderson A R A RKA Chaplain M A J, editor. Single-cell-based models in biology and medicine. Basel: Birkhauser; 2007. p. 107–136.

2. Magno R, Grieneisen VA, Marée AF. The biophysical nature of cells: potential cell behaviours revealed by analytical and computational studies of cell surface mechanics. BMC biophysics. 2015;8(1):8.

3. Albert PJ, Schwarz US. Dynamics of cell shape and forces on micropatterned substrates predicted by a cellular Potts model. Biophysical journal. 2014;106(11):2340–2352.

4. Mori Y, Jilkine A, Edelstein-Keshet L. Wave-pinning and cell polarity from a bistable reaction-diffusion system. Biophysical journal. 2008;94(9):3684–3697.

5. Roux C, Duperray A, Laurent VM, Michel R, Peschetola V, Verdier C, et al. Prediction of traction forces of motile cells. Interface focus. 2016;6(5):20160042.

6. Ng MR, Besser A, Brugge JS, Danuser G. Correction: Mapping the dynamics of force transduction at cell–cell junctions of epithelial clusters. eLife. 2015;4:e06656.

7. Maruthamuthu V, Sabass B, Schwarz US, Gardel ML. Cell-ECM traction force modulates endogenous tension at cell–cell contacts. Proceedings of the National Academy of Sciences. 2011;108(12):4708–4713.

8. Maree et al. (2006) Polarization and movement of keratocytes: a multiscale modelling approach Bulletin of Mathematical Biology. 2006;68:1169–1211.

9. Stein M. Large sample properties of simulations using Latin hypercube sampling. Technometrics. 1987;29(2):143–151.
