# Supplementary figures and images for: From energy to cellular forces in the Cellular Potts Model: An algorithmic approach
Source: PLoS Comput Biol. 2019 Dec 11;15(12):e1007459. doi: 10.1371/journal.pcbi.1007459 (PMC6927661; doi:10.1371/journal.pcbi.1007459)

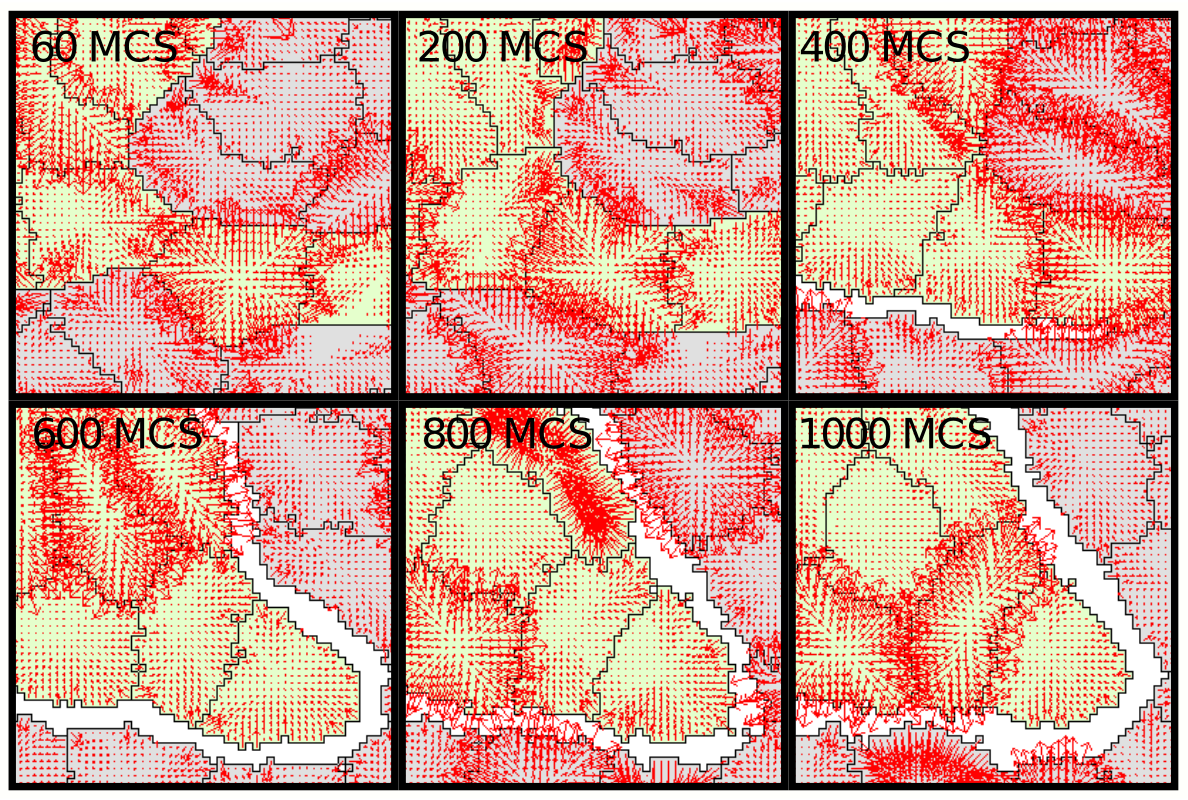

Supplement: S18 Fig — A magnification of the square regions in Fig 9 of the main text. Parameter values were a = 300, λa = 1000, p = 67, λp = 20, J(0, grey) = 1800, J(0, green) = 1800, J(grey, grey) = 900, J(green, green) = 900, J(grey, green) = 9000, ξ(r) = 18, and r = 3 for all neighborhood calculations. The cellular temperature T was set to 600. (PNG) [file pcbi.1007459.s019.png]

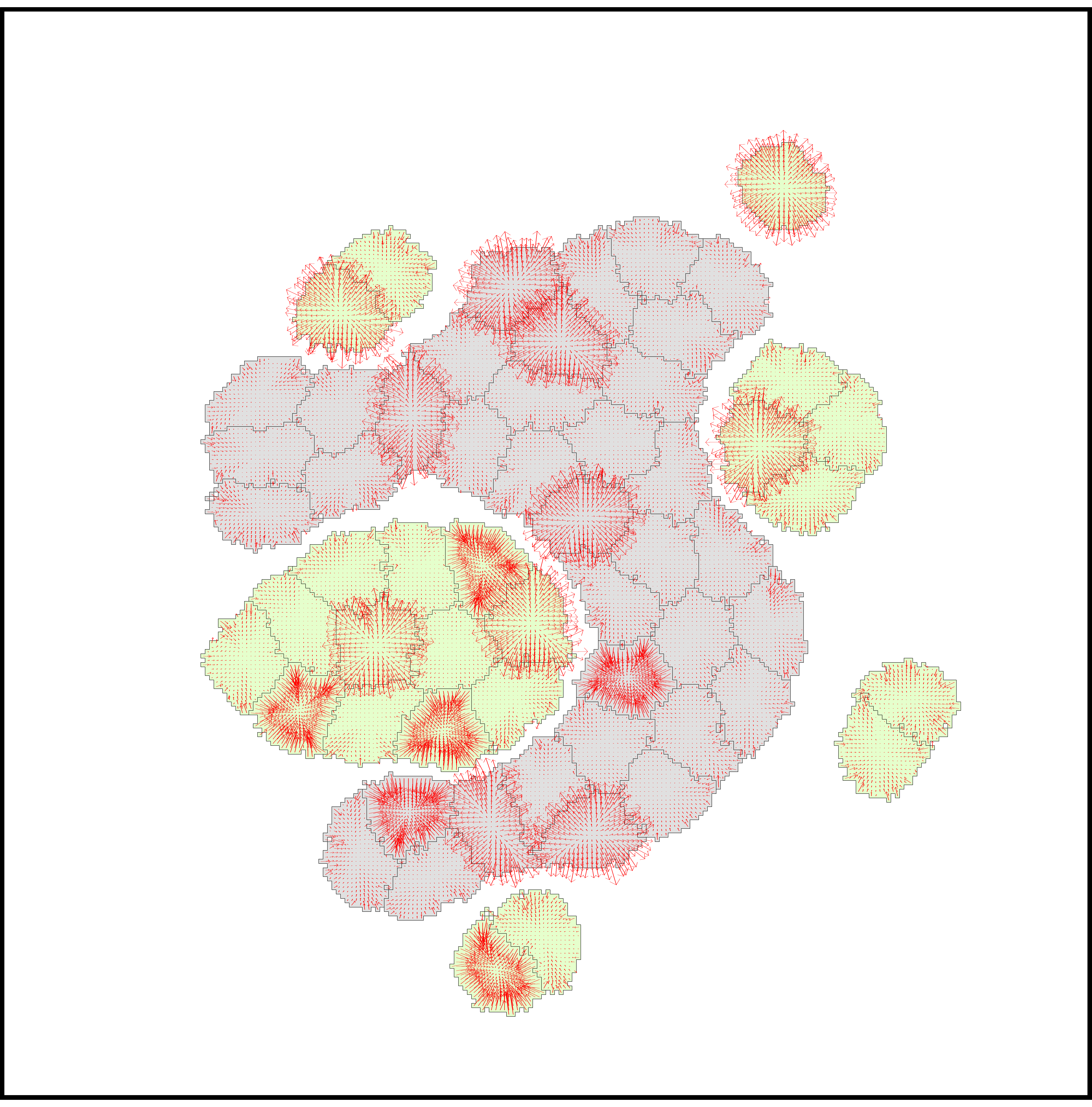

Supplement: S19 Fig — Parameter values were a = 300, λa = 1000, p = 67, λp = 20, J(0, grey) = J(0, green) = 1800, J(grey, grey) = J(green, green) = 900, J(grey, green) = 9000, ξ(r) = 18, and r = 3 for all neighborhood calculations. The cellular temperature T was set to 600. Some cells are still experiencing large forces since the cluster is still not equilibrated. (PNG) [file pcbi.1007459.s020.png]

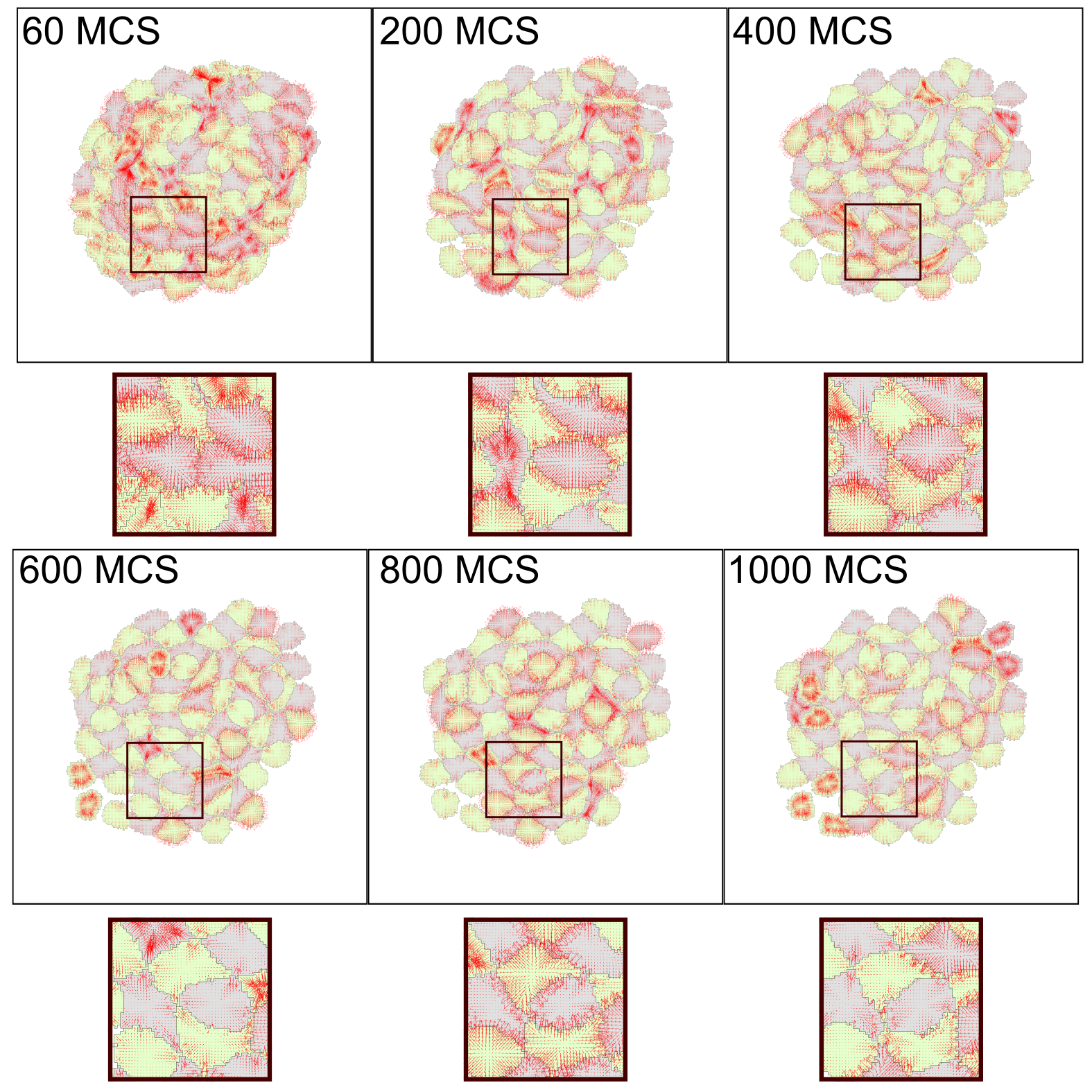

Supplement: S20 Fig — Parameter values were as in S19 Fig but with J(grey, grey) = J(green, green) = 7200, J(grey, green) = 1800. (PNG) [file pcbi.1007459.s021.png]

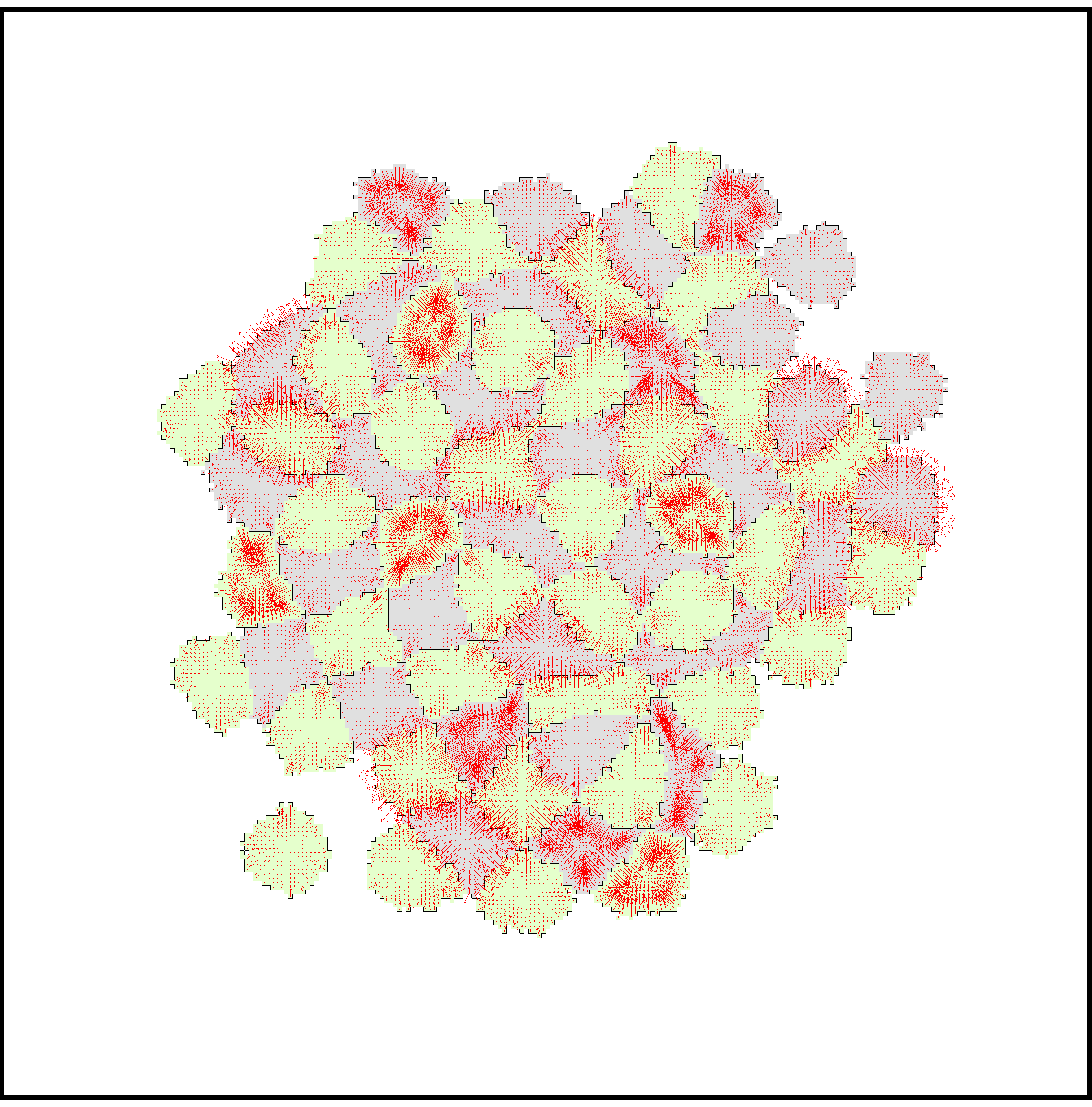

Supplement: S21 Fig — Parameter values were as in S20 Fig. (PNG) [file pcbi.1007459.s022.png]

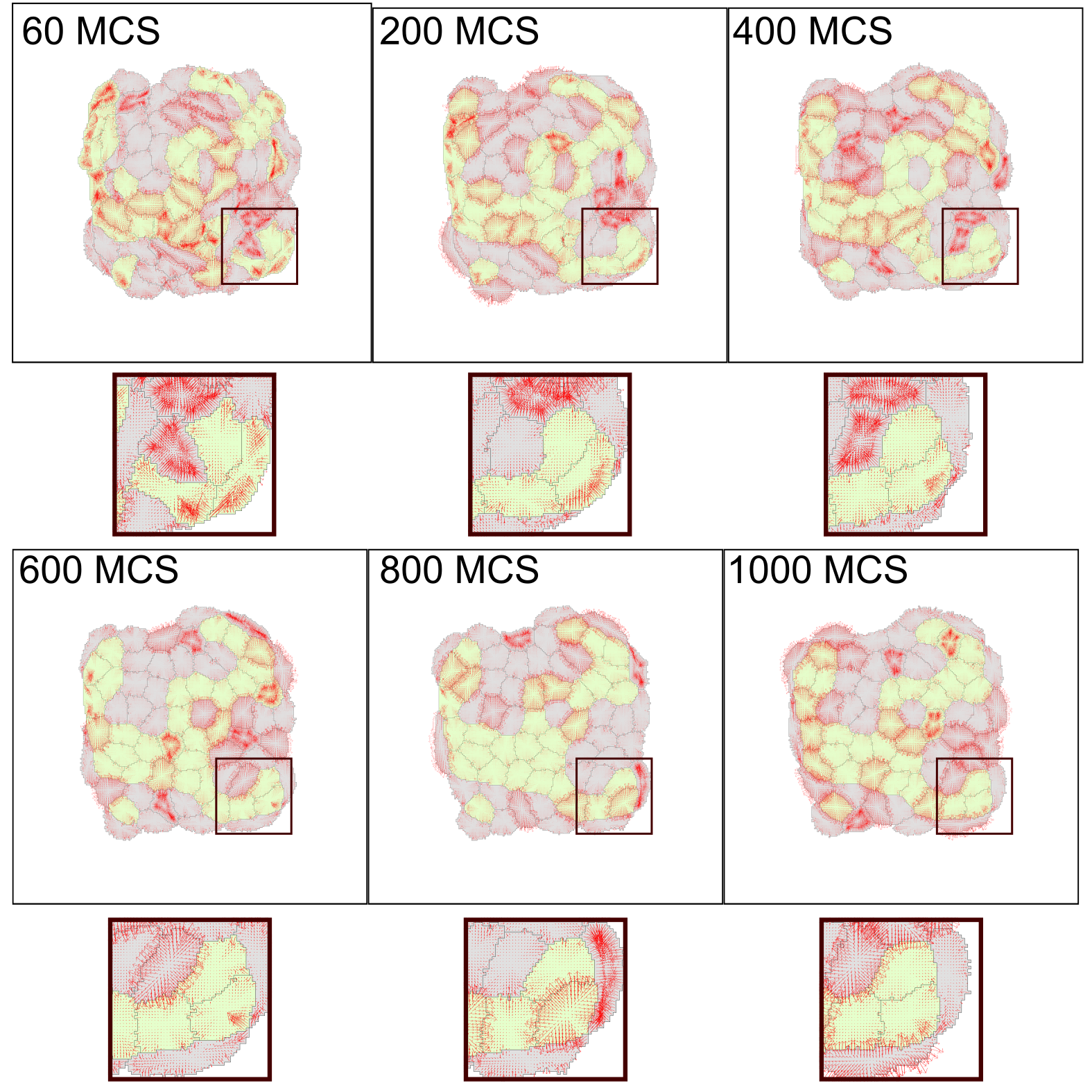

Supplement: S22 Fig — Parameter values were were as in S19 Fig but with J(0, grey) = 1800, J(0, green) = 9000, J(grey, grey) = 1800, J(green, green) = 1800, J(grey, green) = 3600. (PNG) [file pcbi.1007459.s023.png]

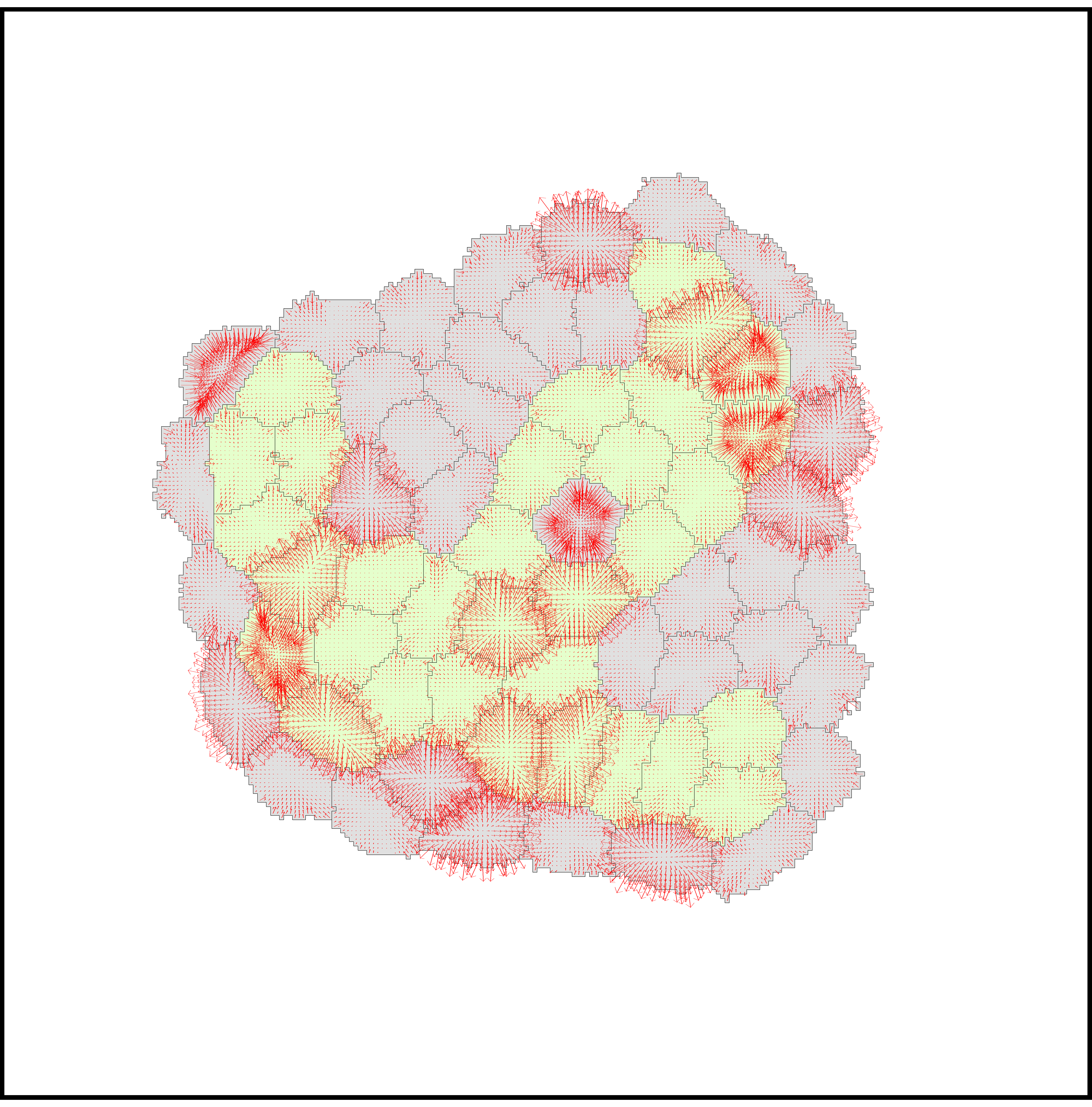

Supplement: S23 Fig — Parameter values as in S22 Fig. (PNG) [file pcbi.1007459.s024.png]
