# Supplementary material for: From energy to cellular forces in the Cellular Potts Model: An algorithmic approach
Source: PLoS Comput Biol. 2019 Dec 11;15(12):e1007459. doi: 10.1371/journal.pcbi.1007459 (PMC6927661; doi:10.1371/journal.pcbi.1007459)
Supplement: S1 Table — Top 5 parameters sets from the Latin hypercube sampling for the round cell, all giving very similar fits. The first set is used in the main text and the force fields for 2-5 are given in S10 Fig. (PDF) [file pcbi.1007459.s025.pdf]

rank	$\lambda_a \cdot \alpha$	$\lambda_p \cdot \alpha$	$J(0,1) \cdot \alpha$	A	P	r
1	0.0278	1.0721	224.8204	888.6	288.9	26.95
2	0.0825	1.2629	135.8610	1567.8	220.41	30.98
3	0.3512	2.8776	423.65	1796.4	248.7	34.3550
4	0.022	1.0243	77.4408	642.3	235.74	32.0450
5	0.1471	0.2469	13.4089	1699.2	256.14	35.64

Table 1: **CPM parameter fits for round cell** Top 5 parameters sets from the Latin hypercube sampling for the round cell, all giving very similar fits.
