# Supplementary material for: From energy to cellular forces in the Cellular Potts Model: An algorithmic approach
Source: PLoS Comput Biol. 2019 Dec 11;15(12):e1007459. doi: 10.1371/journal.pcbi.1007459 (PMC6927661; doi:10.1371/journal.pcbi.1007459)
Supplement: S2 Table — As in Table S1 Table, but for the polarized cell. Multiple parameter sets give very similar fits with SSE around 1.3e6. First set is used in the main text and the force fields for 2-5 are given in S11 Fig. (PDF) [file pcbi.1007459.s026.pdf]

rank	$\lambda_a \cdot \alpha$	$\lambda_p \cdot \alpha$	$J(0,1) \cdot \alpha$	A	P	r
1	0.1347	0.1721	34.1528	849.3	296.52	11.84
2	0.0842	1.0738	153.0155	791.7	292.44	11.2050
3	0.0464	0.3323	46.1646	628.8	280.14	11.43
4	0.0245	0.3430	41.9515	400.5	284.7	11.9350
5	0.1935	0.7110	2.9805	877.5	224.43	11.0750

Table 1: **CPM parameter fits for polarized cell** Top 5 parameters sets from the Latin hypercube sampling for the polarized cell, all giving very similar fits.
